# Rationality: a social-epistemology perspective

**DOI:** 10.3389/fpsyg.2014.00581

**Published:** 2014-06-18

**Authors:** Sylvia Wenmackers, Danny E. P. Vanpoucke, Igor Douven

**Affiliations:** ^1^Faculty of Philosophy, University of GroningenGroningen, Netherlands; ^2^Center for Molecular Modeling, Ghent UniversityGhent, Belgium

**Keywords:** social epistemology, rationality, computer simulations, opinion dynamics, beliefs, theory, inconsistency, probability

## Abstract

Both in philosophy and in psychology, human rationality has traditionally been studied from an “individualistic” perspective. Recently, social epistemologists have drawn attention to the fact that epistemic interactions among agents also give rise to important questions concerning rationality. In previous work, we have used a formal model to assess the risk that a particular type of social-epistemic interactions lead agents with initially consistent belief states into inconsistent belief states. Here, we continue this work by investigating the dynamics to which these interactions may give rise in the population as a whole.

## 1. Introduction

This paper aims to show the importance of a social perspective in the study of human rationality. While, as will be seen, the work we present relies on computer simulations, we believe it may inspire further empirical research by social scientists. Computer simulations, such as those to be presented, form a bridge between normative models and descriptive results. The simulations depend on a theoretical model with various parameters. Some combinations of the parameters may be optimal for the attainment of one or more norms, whereas other combinations of parameters may give a good approximation to an epistemic group of real people. If the model parameters can be linked to variables in the real world, this may enable us to give practical advice for increasing rationality in social settings.

In previous work, we studied a formal model of a type of epistemic interactions in which agents whose belief states are in some sense close together compromise by settling on a kind of “averaging” belief state. We showed that compromising in this way carries the risk of leading agents with initially consistent belief states to become inconsistent. Although it was shown in the same paper how this risk could be minimized, it might nonetheless be considered as a reason for banishing the designated kind of interactions. Here, we continue the previous work by investigating the dynamics of a population as a whole to which epistemic compromising may give rise. We pay special attention to the conditions under which such compromising may lead to a consensus among the members of a population. This is intended to shed new light on the question of whether it is at all rational to interact epistemically in the said kind of way.

## 2. Theoretical background

In their study of human rationality, philosophers as well as psychologists of reasoning have tended to focus on individual thinkers in isolation from their social environment. Which beliefs an individual ought to hold and how an individual ought to change his beliefs have traditionally been regarded as questions that are independent of which beliefs other individuals hold or how other individuals change their beliefs. This is at least somewhat surprising, given that we are so obviously members of a community of individuals who pursue by and large the same epistemic goals, who frequently engage in common activities to gather new evidence, who constantly exchange information, who often (have to) rely on the words of others, who regularly seek each other's advice in epistemic matters, and who sometimes put great effort into trying to influence one another's opinions. In fact, we see these kinds of behavior not just in everyday life, but also, and even especially, in the practice of science, which many regard as producing the—in some sense—best and most valuable knowledge. Doubtlessly, there are more and less rational ways of engaging in these various activities, and it would seem part of the business of philosophy, as well as of that of psychology, to sort out which are which.

At least in philosophy, there is a growing awareness that the general neglect of the group level in studying human rationality has created a serious gap in our understanding indeed, and philosophers have begun to correct this lacuna^1^. Their efforts have given rise to a field now commonly known as “social epistemology” (Goldman, 1999). Questions addressed so far by social epistemologists concern the possibility of testimonial justification (in particular, the question of whether we are justified in holding a belief on the basis of another person's testimony; see Douven and Cuypers, 2009, Fricker, 1987, and Lackey, 1999), the rationality (or otherwise) of aligning our opinions on a given matter with those of experts on the matter (Gaifman, 1986; van Fraassen, 1989, Ch. 8; Goldman, 2001), and the effect on our beliefs that the discovery of peer disagreement should have (that is, the question of whether we can rationally stick with our belief after the discovery that someone who we regard as a peer holds a contrary belief; see, for instance, Douven, 2009, 2010, and Elga, 2007).

The popularity of social epistemology being on the rise, it is easily missed that we still do not know whether, on balance, the possible benefits of social-epistemic interactions outweigh their possible costs. Indeed, various philosophers and also sociologists have enthusiastically reported about the “wisdom of the crowds,” in the context of which it has been asserted that the aggregated opinions of a group of laypeople is often closer to the truth than the opinions of individual experts (Surowiecki, 2004). Such assertions might make one forget that crowds can be wildly erratic and irrational, too. We know how the crowd responded when, in his *Sportpalast* speech in February 1943, the Nazi minister of propaganda Joseph Goebbels asked whether it wanted total war. There was little wisdom in that response^2^.

In trying to give cost–benefit analyses of diverse types of social epistemic interactions, and also for related purposes, a number of social epistemologists have recently started using computer simulations for studying communities of epistemically interacting artificial agents, where the agents typically adapt their beliefs (fully or partially) on the basis of information about the beliefs of other agents in the community. It has been argued that, insofar as these methods capture central aspects of the epistemic interactions between *real* agents, they give important information about the conduciveness of these interactions to the achievement of our epistemic goals as well as about the costs that may come with the interactions.

By far the most research on rationality is concerned either with developing an experimentally informed descriptive model of actual human thinking or with developing a theoretically-oriented normative model of idealized human thinking. We present a study that nominally falls in the first category, in that we study opinion dynamics with the help of computer experiments concerning epistemically interacting agents. But it would be more accurate to say that our study falls somewhere on the continuum between descriptive and normative work. The agents that we model are inspired by particular aspects of human thinking (such as the observation that humans have opinions on multiple topics, some of which are logically independent, and some of which are logically connected) and human epistemic interaction (most notably, that in practice we allow others' beliefs to influence our own as well as try to make our beliefs influence those of others), but—as will emerge—they clearly lack other characteristics of human thinking. So, it is not a purely descriptive study. The agents in the simulations do follow a prescribed way of revising their opinions and never fail to adhere to it, but they are still non-ideal thinkers; for example, they may come to believe an inconsistency without realizing this. Hence, it is not a purely normative model either.

As mentioned in the introduction, we here continue work that we have started elsewhere (Douven, 2010; Douven and Riegler, 2010, and especially Wenmackers et al., 2012). Specifically, we present a formal model for studying a community of agents that update their belief states by “averaging” (in a certain well-defined sense) over the belief states of agents that are close enough to their own belief state (where “close enough” will also receive a precise definition). In Wenmackers et al. (2012), we studied the question of how probable it is that averaging (in the designated way, yet to be specified) over others' belief states leads one into a state of inconsistency. Here, we investigate the opinion dynamics in a more global way: we consider the entire epistemic space (not just those situations in which some or all of the agents arrive at an inconsistency) and we do not restrict the dynamics to a single step (although it turns out that for the examples we consider, all of the dynamics plays out in just two steps). This approach gives us new insights into the previously obtained results and it allows us to visualize the process that the community as a whole undergoes as a result of the updates by its members. As stated in the introduction, we will be especially interested in the conditions under which the social-updating process leads to a consensus among the members of the community (including consensus on the inconsistent theory)^3^. We will give a brief summary at the end of each section. For a quick overview of the article, the reader may skip forward to these paragraphs of key points.

## 3. The model

The most widely known formal model for studying the effects of epistemic interactions on the belief states of individual agents is probably the model developed in Hegselmann and Krause (2002, 2005, 2006), which now generally goes by the name of “Hegselmann–Krause model” (“HK model,” for short). This model has received attention from researchers from various quarters, including philosophers, mathematicians, social scientists, and physicists (see, e.g., Deffuant et al., 2000; Dittmer, 2001; Weisbuch et al., 2002). It has also been used mainly to investigate descriptive questions, such as the question under which conditions the opinions of interacting agents are likely to polarize and under which conditions these opinions are likely to converge, but it has been used to investigate some normative issues as well (see Riegler and Douven, 2009; Douven, 2010). We consider a variant of the HK model. First, we present our general framework. In the course of this section, we present two examples. (Some readers may find it beneficial to consult the examples 3.1 and 3.2 prior to reading the more abstract setup.)

The basic version of the HK model assumes communities of agents that are trying to determine the value τ of some unspecified parameter by repeatedly and simultaneously averaging over the opinions of those agents that are within their so-called Bounded Confidence Interval (BCI)^4^. One agent is in a second agent's BCI—or, as we shall sometimes say, following Douven (2010), is a second agent's (epistemic) peer—precisely if the absolute difference between their opinions about the value of τ does not exceed some given threshold value ϵ. Hegselmann and Krause also study a model in which the agents take into account evidence about τ that they receive “directly from the world.” More exactly, in this model the opinion of agent *x_i_* after the (*u* + 1)-th update is given by

(HK) $$x_{i}(u+1)=\alpha \frac{1}{\left|X_{i}(u)\right|}\sum_{j\in X_{i}(u)}x_{j}(u)+(1-\alpha )\tau ,$$

where *x_i_*(*u*) is the opinion of agent *x_i_* after the *u*-th update, whose peers (agents within the BCI after the *u*-th update) form the set $X_{i}(u):=\{j:|x_{i}(u)-x_{j}(u)|⩽\epsilon \}$ and α ∈ [0, 1] is the relative importance of the social-updating process as compared to evidence-gathering. In the basic version of the HK model, without evidence-gathering, α = 1.

It is a limitation of the HK model that it considers only agents whose belief states consist, at any given point in time, of just one value. In Riegler and Douven (2009), an extension of the HK model was proposed that allows agents to have richer belief states in that they have beliefs on different aspects of the world. In other words, each agent holds a theory about the world, where a theory consists of a set of propositions expressible in the agent's language. A theory may be consistent or inconsistent: if no world can satisfy all the agent's beliefs—for instance, as when an agent believes that snow is white and also believes that snow is not white—then the agent holds an inconsistent theory about the world; otherwise the theory is consistent. Note that consistency does not guarantee truth: it may happen that some world or worlds satisfy all the agent's belief, but that the actual world does not. However, inconsistency does guarantee falsity: if a theory is true of no world—no world satisfies all of the agent's beliefs—then a fortiori it is not true of the actual world. Agents' belief states are supposed to be closed under (classical) logical derivability, meaning that any proposition expressible in the agent's language that follows logically from the agent's theory ipso facto belongs to that theory. As a result, the theory an agent holds can be represented by the strongest proposition it implies.

Given *M* atomic propositions, there are *w*_*M*_ = 2^*M*^ possible worlds that we can distinguish between. In turn, this means that there are *t*_*M*_ = 2*^w_M_^* theories about the world, exactly one of which represents the inconsistent theory, in which all the possible worlds have been ruled out by the agent. There is also exactly one tautology, the theory in which all possible worlds are left as epistemic possibilities for the agent. Note also that, by assuming some ordering of the possible worlds, the belief state of each agent can be represented by a bit string, where a 1-bit (0-bit) at the *n*-th location indicates that world number *n* (in the given ordering of worlds) is deemed possible (impossible) by the agent^5^.

In this model, agents revise their theory of the world by taking into account the theories held by certain other agents in the community, comparable to how the agents in the HK model update. However, now the BCI is defined in a slightly more complicated way. To quantify the distance between two theories, the so-called Hamming distance δ between the corresponding bit strings is used: this distance is given by the number of locations in which these strings differ. The BCI is then defined by placing a threshold value *D* for δ, meaning that in updating the agents take into account the belief state of another agent if, and only if, the Hamming distance between (the bit string representing) the agent's own theory and (the bit string representing) the other agent's theory is smaller than or equal to *D*. An example may help to make this less abstract.

**Example 3.1**. Consider an interpreted propositional language 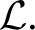 with just two atomic propositions, *p*, expressing that snow is white, and *q*, expressing that grass is green. Then there are 2^2^ possible worlds: the world in which *p* and *q* both hold, the world in which *p* holds but *q* does not, the world in which *q* holds but *p* does not, and the world in which neither *p* nor *q* holds. Let these worlds be ordered in this way, so that the world in which both *p* and *q* hold is world number 1, and so on. Then the 16 theories that can be formulated in 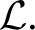 can be coded as 4-digit strings. For example, the string 1111 codes the tautology: the actual world corresponds to one of the four possible worlds; the string 0000 codes the inconsistent theory: the actual world corresponds to none of the four possible worlds; and 1100 codes the theory according to which snow is white and grass may or may not be green. Finally, if one agent holds the theory 1100 and another agent holds the theory 1001 (the theory according to which the world is such that *either* snow is white and grass is green *or* snow is not white and grass is not green), then the Hamming distance δ between their theories (that is, between the bit strings representing these theories) equals 2, given that they differ in the second and fourth bit and coincide otherwise.       ◊

The update rule for theories in this model—so, basically the analog of (HK)—is a bitwise operation in two steps: (1) averaging and (2) rounding. In step (1), for each bit of the theory, a straight average is taken of the corresponding bit of those agents that are within the agent's BCI (note that this includes the agent himself). In general, the result is a value in the interval [0, 1] rather than just a 0 or 1. Hence the need for step (2): in case the average is greater than 1/2, the corresponding bit is updated to 1; in case the average is less than 1/2, the corresponding bit is updated to 0; and in case the average is exactly equal to 1/2, the corresponding bit keeps its initial value.

More formally, the *n*-th bit of the bit string representation of agent *x_i_*'s belief state after the (*u* + 1)-th update as determined by the extended HK update rule is

(EHK) $$x_{i}(u+1)[n]=\{\begin{array}{ll} 1 & \text{if}\frac{\text{1}}{\text{|}X_{i}(u)\text{|}}\sum_{j\in X_{i}(u)}x_{j}(u)[n]>\frac{\text{1}}{\text{2}},\\ 0 & \text{if}\frac{\text{1}}{\text{|}X_{i}(u)\text{|}}\sum_{j\in X_{i}(u)}x_{j}(u)[n]<\frac{\text{1}}{\text{2}},\\ x_{i}(u)[n] & \text{otherwise,} \end{array}$$

with the set of peers of agent *i* after the *u*-th update now $X_{i}(u):=\{j:\delta (x_{i}(u),x_{j}(u))\;⩽D\}$. Actually, in Riegler and Douven (2009) the agents also obtained evidence from the world, more or less as in one of the versions of the HK model. However, in our Wenmackers et al. (2012) we considered only the more basic (EHK), as we will do here. In Wenmackers et al. (2012) and also in the present paper, it is assumed that the agents update their beliefs simultaneously and repeatedly, at discrete time intervals. We again give an example.

**Example 3.2**. Consider a community of nine agents that share our earlier language 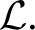 Let the bit string representations of their initial belief states be

**Table:** 

1. 1100	4. 1000	7. 0000
2. 1101	5. 1101	8. 1101
3. 0001	6. 0001	9. 0001

Assume that *D* = 1. Then, for instance, the set of peers of agent 1 is initially (after 0 updates): *X*_1_(0) = {1, 2, 4, 5, 8}. Agent 1 will update his theory to *x*_1_(1) = 1101, given that all agents in *X*_1_(0) deem the first world possible, and hence *x*_1_(1)[1] = 1; all but one of the peers deem the second world possible, so *x*_1_(1)[2] = 1; all peers deem the third world impossible, so *x*_1_(1)[3] = 0; and although *x*_1_ initially deems the fourth world *im*possible, all other agents in *X*_1_(0) deem that world *possible*, and so *x*_1_(1)[4] = 1.       ◊

In Wenmackers et al. (2012), we computed the probability for an agent with a consistent belief state to arrive at an inconsistent belief state after a single update via (EHK). Except for the trivial cases with *N* = 2 or *D* = 0, we found that the probability of this event happening is always higher than zero, but lower than 2%. Moreover, we formulated some practical suggestions to avoid arriving at the inconsistent theory. For instance, it was shown that including more independent properties (increasing *M*) lowers the probability. Also, the members of even-numbered groups of agents (*N* even) have a lower probability of updating to the inconsistent theory than have the members of odd-numbered groups of comparable size. And the BCI was shown to play an important role, too: low threshold values *D* (narrow BCIs) result in low dynamicity, so the probability of any change in belief state is low, so a fortiori the probability of arriving at an inconsistency is low; very high bounds of confidence (*D* close to 2^*M*^) were also shown to decrease the chance of updating to the inconsistent theory.

The mere possibility of arriving at an inconsistent theory—even though it has a low probability—might be thought to discredit EHK. But this would be to overlook that the update rule can have compensating advantages. The extension of the HK model that was studied in Riegler and Douven (2009) was in that paper shown to offer a clear advantage over “individualistic” updating in cases where the agents received evidence that is to some extent noisy (as evidence typically is); in such cases, the social updating led agents to approach the true theory more closely in a shorter time span. That already the simpler update rule (EHK) may offer advantages can be seen by considering agent number 7 in Example 3.2. This agent initially holds the inconsistent theory but after updating comes to hold a consistent theory. (One easily verifies that *X*_7_(0) = {3, 4, 6, 7, 9} and that averaging-and-rounding over the corresponding belief states results in a consistent belief state, to wit, *x*_7_(1) = 0001.) However, to give a more systematic answer to the question of which advantages updating via (EHK) may have, more must be known about the properties of this update rule.

To take further steps toward determining which properties (EHK) has, beyond the ones presented in Wenmackers et al. (2012), the remainder of this paper considers this update rule again as used by a group of *N* agents whose belief states are theories of the world concerning *M* binary properties. However, now we focus our attention on the process of updating via (EHK) *repeatedly*. We achieve this by investigating the structure of the “belief space” as a whole. Due to the update rule, and starting out from a particular belief state (or theory of the world), some belief states can be reached in a single step, whereas other belief states can only be reached via intermediate steps, or cannot be reached at all. So, perhaps a larger portion of the agents will reach the inconsistent theory after repeated updating. On the other hand, agents that start out from the inconsistent theory may leave it afterwards (as just seen). *A priori*, it is not clear whether the probability of reaching the inconsistent theory after a single time step is an under- or an overestimation of the probability of reaching the inconsistent theory in general. It is good to keep in mind that, ultimately, we are not interested in estimating this probability for the model *per se*. Rather, we aim to identify useful parameters to lower the probability of arriving at inconsistencies in actual human thinking, or to escape them once they have occurred.

Our investigations in the following focus on the case in which there is only one binary property that the agents consider to form their theory about the world (i.e., *M* = 1). In this case, there is one proposition, which can be true or false, so there are two possible worlds. There are four theories: 00 (the inconsistent theory), 01, 10, and 11 (the tautology). The Hamming distance between two different theories is either 1 (between 00 and 01, between 00 and 10, between 01 and 11, and between 10 and 11) or 2 (between 00 and 11 and between 01 and 10). It may be argued that studying *M* = 1 defeats the original purpose of modeling agents that hold theories. After all, we introduced theories of the world as a means to study agents with rich belief sets. If there is only one binary property of interest to the agents, it seems overly complicated to consider theories. Nevertheless, *M* = 1 is an important case from the theoretical viewpoint, because the relevant dynamics can be represented in three dimensions, whereas higher values of *M* correspond to higher-dimensional spaces, which makes it harder to visualize them. Moreover, some of the conclusions that can be reached for the *M* = 1 toy model do generalize to the higher-dimensional case. We give a brief, qualitative discussion of the general case at the end of this article.

### 3.1. Key points

We model a group of *N* agents. Their opinions concern *M* binary properties of the world. There are *t_M_* = 2^*M*^ possible worlds (or combinations of the properties being true or false in the world). Each agent holds a theory about the world, which can be represented as a string of *t_M_* bits, where zero means that the agent has ruled out the corresponding possible world. There are 2^*t_M_*^ such theories. Agents consider as epistemic peers those agents who currently hold a “sufficiently similar” theory, which means that the number of bits that are different between the agent's own theory and that of a potential peer is less than a certain threshold, called the bound of confidence *D*. Agents adjust their theory by averaging over the theories held by their peers. We study the resulting opinion dynamics.

## 4. Opinion-profile space

**Figure 1:**
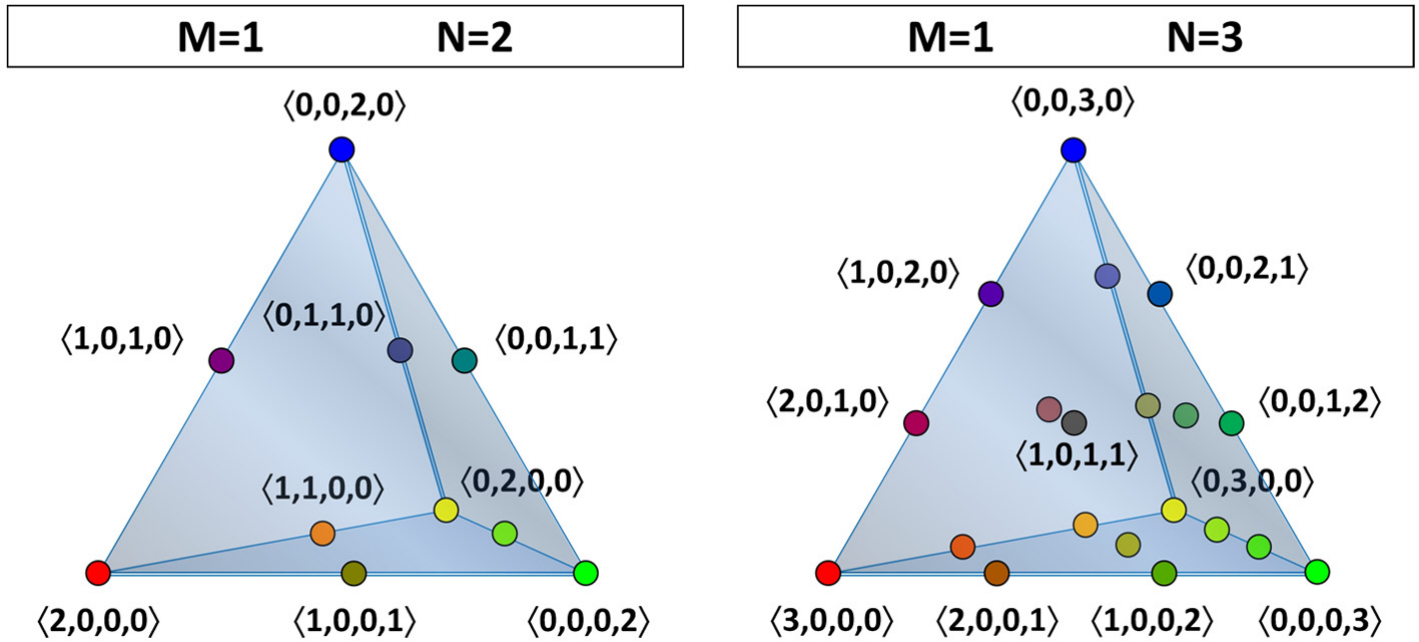
**The belief space or opinion-profile space (OPS) for the theories in a language with one atomic proposition (*M* = 1) can be visualized as a discrete grid in a tetrahedral volume**. The number of grid points depends on the population size. The OPS of a population of two agents is shown at the left (*N* = 2), while that of three agents is shown at the right (*N* = 3). The grid points are indicated by colored dots and are labeled by their opinion-profile coordinates between angle brackets. (For *N* = 3, only the four vertices and the opinion profiles located in the front face are labeled.)

Our goal is to investigate how the opinions in the population as a whole change over time due to the iterated application of (EHK) by the individual agents. To achieve this, we first need to identify the relevant belief space, by which we mean the phase space in which we can represent the opinion dynamics of the entire group of agents. An *opinion profile* is a vector $\vec{n}$ that specifies how many agents in the entire population occupy each of the belief states (at a given point in time). In general, $\vec{n}$ has *t*_*M*_ components, which sum to *N*. (Unlike Example 3.2, the opinion profile is anonymous, so it does not keep track of which agent holds which theory.) The relevant belief space is what we will call the “opinion profile space” (OPS), in which each point represents a possible opinion profile. For *M* = 1, opinion profiles have four components, 〈*n*_00_, *n*_01_, *n*_10_, *n*_11_〉, which can be represented in a three-dimensional tetrahedron. For a representation of the tetrahedral OPS with two (*N* = 2) or three agents (*N* = 3), see Figure 1.

To elaborate, if there are two agents (*N* = 2), then there are ten different opinion profiles. In other words, the OPS consists of ten points, which are shown at the left-hand side of Figure 1. Four of these ten opinion profiles represent a consensus: 〈0, 0, 0, 2 〉, in which the two agents agree on theory 11; 〈 0, 0, 2, 0〉, in which the two agents agree on theory 10; 〈0, 2, 0, 0〉, in which the two agents agree on theory 01; and 〈2, 0, 0, 0〉, in which the two agents agree on theory 00. The remaining six points in the OPS represent opinion profiles in which each agent holds a different position: 〈0, 0, 1, 1〉, in which one agents holds theory 11 and the other holds 10; 〈0, 1, 0, 1〉, in which one agents holds theory 11 and the other holds 01; and so on. Thus, in the case with *M* = 1 and *N* = 2, the only points that can be occupied in the OPS are the four vertices of a tetrahedron (consensus) and the six midpoints of the edges (disagreement).

If there are three agents (*N* = 3), then there are twenty different opinion profiles, corresponding to an OPS that consists of twenty points, as can be seen on the right-hand side of Figure 1. There are still four possible opinion profiles that represent a consensus—〈0, 0, 0, 3〉, 〈0, 0, 3, 0〉, 〈0, 3, 0, 0〉, and 〈3, 0, 0, 0〉—corresponding to the vertices of the tetrahedral OPS. There are twelve profiles in which two agents agree and the third one does not: two on each of the six edges in the OPS. And there are four ways in which all of the agents can disagree with each other; these opinion profiles each correspond to a point on one of the four faces of the OPS.

For any fixed number of *N* agents (and some number *M* of propositions) the opinion profile space is discrete and contains $\frac{(N+t_{M}-1)!}{N!(t_{M}-1)!}$ points (this is the (hyper-)tetrahedral number of order *N* + 1 in *t*_*M*_ − 1 dimensions, or the multiset coefficient of choosing *N* times with repetition out of *t*_*M*_ options). If there are four or more agents, then the points in the OPS also occupy the interior volume of the tetrahedron. (For four agents, this concerns only the central point 〈1, 1, 1, 1〉).

In principle, the OPS for any particular *N* can be computed by hand: for each possible opinion profile, one can determine each agent's peer group and apply the two-step update rule. In practice, however, a computer is required to assist in these computations, since the aforementioned number of opinion profiles in the OPS grows rapidly with *N*. To this end, we have written a program in Object Pascal. Instead of iterating the process for each opinion profile until it reaches a fixed point, we instructed the program to link up opinion profiles that reach a fixed point, via intermediate opinion profiles. In section 5, we will show how to abstract from the number of agents in the population (by looking at the opinion density instead of the opinion profile), but first we introduce the dynamics on the OPS brought about by social updating via (EHK).

### 4.1. Results: dynamics on the opinion profile space

We view the OPS equipped with the two-step social update rule (EHK) (with *N* agents and a threshold value *D*) as a discrete dynamical system. Even before we look at the results, we can give a qualitative description of the dynamics. For any value of *D*, certain opinion profiles will act as fixed points. Populations that start out with an opinion profile outside a fixed point may be driven either toward a certain fixed point (“sink,” or stable equilibrium, or attractor) or away from it (“source” or unstable equilibrium). All unstable points that are attracted toward a particular sink belong to the “basin” of this sink.

The lower the threshold *D*, the more fixed points we expect to find in the OPS. In the case with *D* = 0, there is no dynamics at all: the agents do not take into account any other opinions, so there is no process of social updating, and all the points in the OPS act as fixed points. (Since there is no dynamics, we cannot classify the points as sources or sinks; rather, this is a case of indifferent equilibrium.) As the BCI increases, an growing number of other opinions may be taken into account and fewer opinion profiles are fixed points. When the BCI is maximal (i.e., *D* = *t_M_*), the dominant sources and sinks are revealed. Opinion profiles in which the agents all agree on the same theory are sinks.

**Figure 2:**
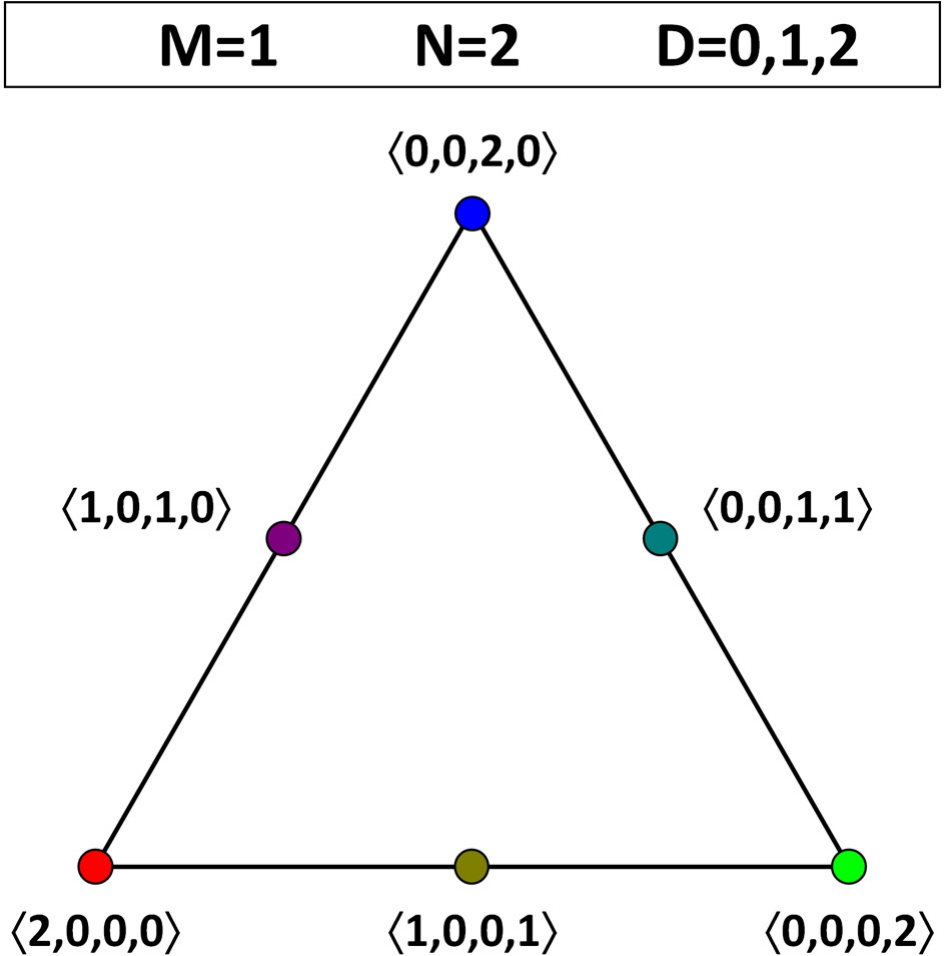
**Opinion dynamics for theories in a language with one atomic proposition (*M* = 1) and a population consisting of two agents (*N* = 2)**. One face of the OPS is shown. Irrespective of the value of the threshold *D* (*D* is equal to 0, 1, or 2), all opinion profiles are fixed points (including the ones not shown).

We will represent the dynamics on the OPS by arrows that point from an initial opinion profile toward the corresponding final state. For the sake of illustration, we consider populations in which none of the agents hold theory 01, so that we can limit ourselves to one face of the OPS. First, suppose that there are just two agents. In this case, there is no dynamics, irrespective of the value for *D*. (After all, when the average is exactly equal to 1/2, the corresponding bit keeps its initial value. Hence, an agent can never be swayed by a single peer and vice versa.) This situation is illustrated in Figure 2: all the opinion profiles are fixed points, so there are no arrows connecting any of them.

**Figure 3:**
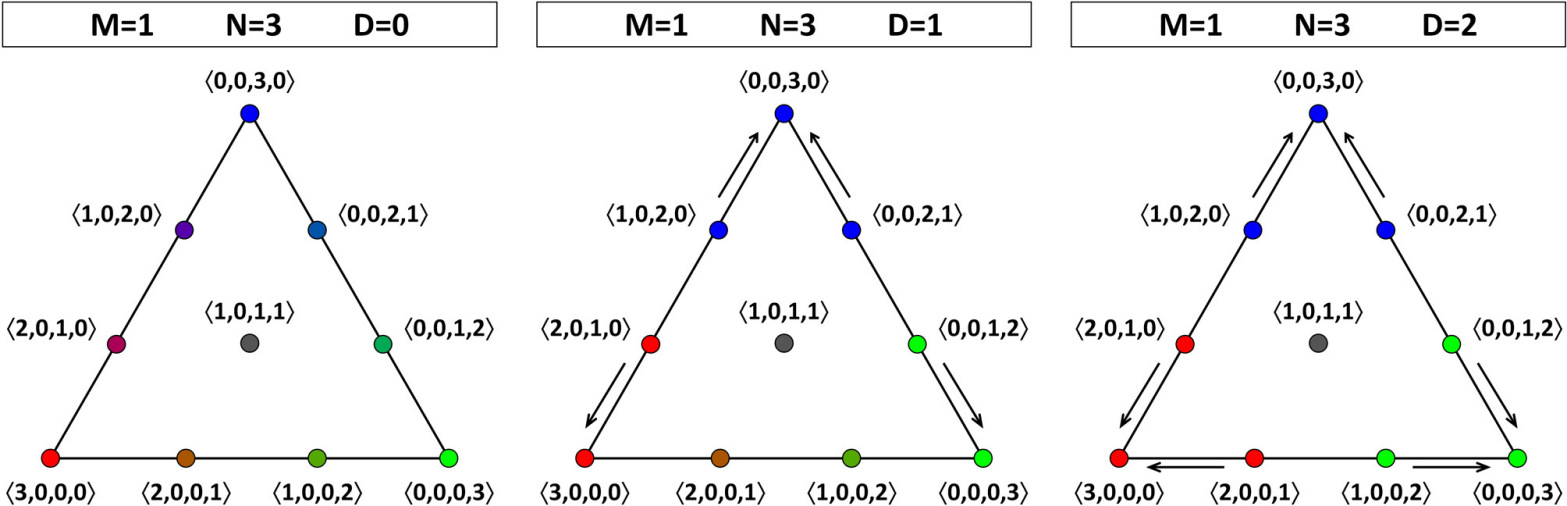
**Opinion dynamics for theories in a language with one atomic proposition (*M* = 1) and a population consisting of three agents (*N* = 3)**. One face of the OPS is shown. For *D* = 0 (left), there is no dynamics; all opinion profiles are fixed points. For *D* = 1 (middle), the three vertices act as sinks, but the points on the lower “double” edge (connecting profiles with a consensus on “more distant” theories—see the main text for details) show a different dynamics than those on the two “single” edges. For *D* = 2 (right), the three vertices act as sinks, and the asymmetry between points on the edges is removed.

**Figure 4:**
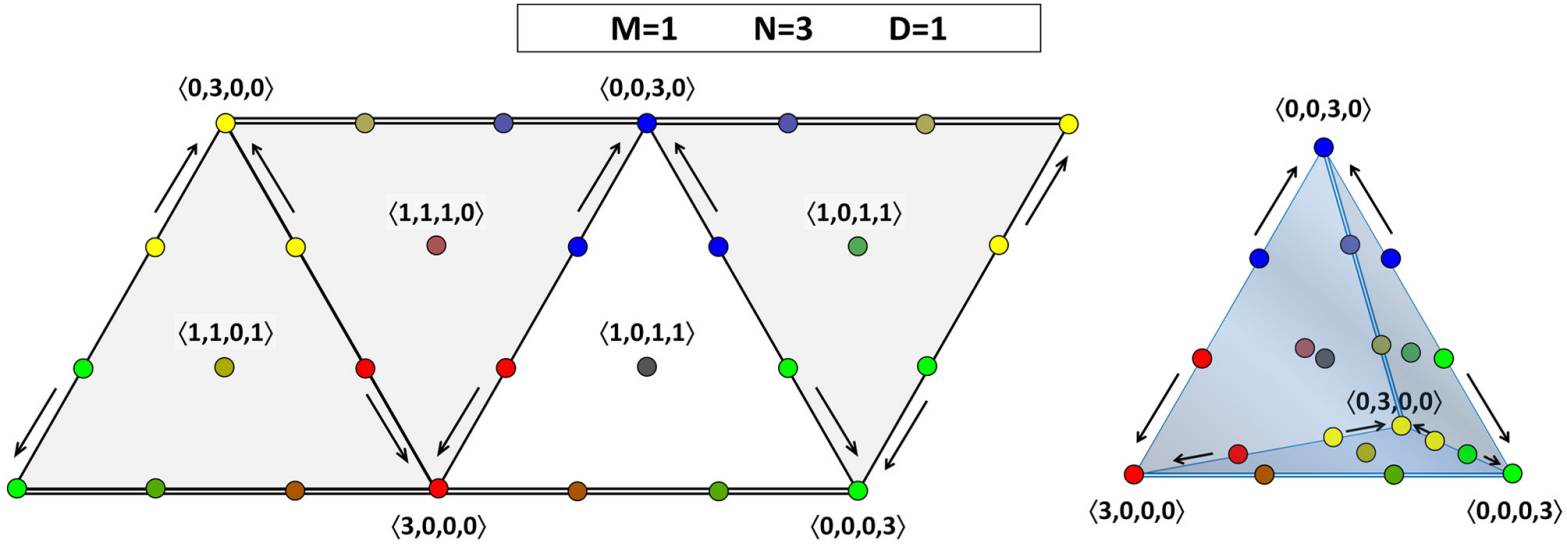
**Opinion dynamics for theories in a language with one atomic proposition (*M* = 1) and a population consisting of three agents (*N* = 3) for *D* = 1**. On the left-hand side, all the faces of the tetrahedral OPS are shown side by side. The face from Figure 3 is shown in white, the others in gray. Observe that the outer edges and their vertices are shown multiply: the leftmost diagonal edge is equal to the rightmost diagonal edge, and the left horizontal edge at the top (bottom) is the mirror image of to right horizontal edge at the top (bottom). On the right-hand side, the tetrahedral OPS is shown. This three-dimensional view allows us to verify that each vertex attracts two other opinion profiles.

If there are three agents, then for *D* = 1 and *D* = 2 there is some dynamics: Figure 3 shows us that the consensus positions (at the vertices) act as sinks. For *D* = 1, there is a certain asymmetry in the face of the OPS that we are considering: there are two opinion profiles that move toward the consensus position at 〈0, 3, 0, 0〉, but only one opinion profile each that moves toward the consensus positions at 〈3, 0, 0, 0〉 and 〈0, 0, 0, 3〉. To understand why not all directions in the tetrahedron are equivalent, we have to remember that there are two pairs of theories that have a larger Hamming distance between them than the other six pairs, one pair being 00 and 11, the other pair being 01 and 10. Therefore, also the two edges connecting opinion profiles corresponding to a consensus on such a pair of “more distant” theories are qualitatively different from the other six edges. In Figure 4, the two edges connecting consensus on “more distant” theories are indicated by a double line, whereas the four other edges are represented by a single line. Since each face has two “single” edges and one “double” edge, the analysis of each of the four faces is equivalent. The right-hand side of Figure 4 also illustrates that the four vertices are equivalent in the sense that they all attract two other opinion profiles (for *D* = 1). The asymmetry between the edges of a single face that appeared for *D* = 1 is absent for *D* = 2, where each sink attracts two other points (at least on the face that we are considering; it attracts three points in total). The explanation for this restoration of symmetry is that, with the maximal value for *D*, even agents that hold maximally different theories regard each other as peers. So, unlike for *D* < 2, they do influence each other in updating their belief states. The point at the middle of the face, 〈1, 0, 1, 1〉, is a non-attracting fixed point (source).

**Figure 5:**
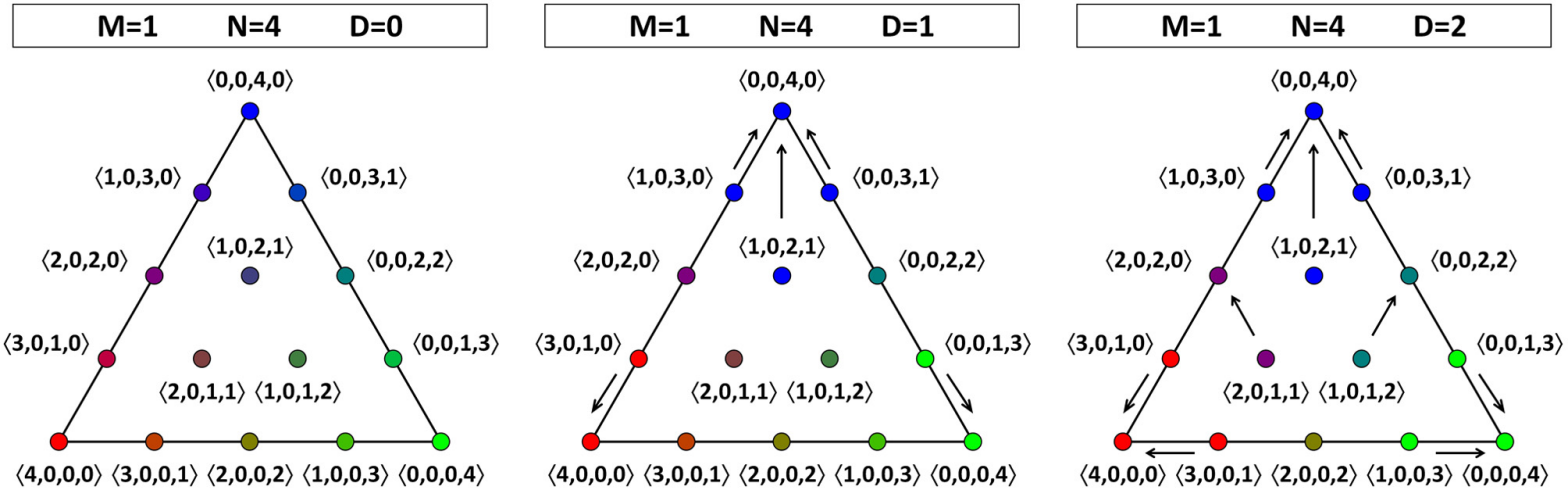
**Opinion dynamics for theories in a language with one atomic proposition (*M* = 1) and a population consisting of four agents (*N* = 4)**. We show the same face of the OPS for *D* = 0 (left), *D* = 1 (middle), and *D* = 2 (right).

Figure 5 shows the opinion dynamics for a population of four agents. Although there are more points in this OPS, the results are comparable to those for *N* = 3: there is no dynamics for *D* = 0, and there is an asymmetry for *D* = 1 that is absent for *D* = 2. The three vertices are sinks and each of the three points at the middle of an edge is a source.

So far, we have considered the opinion dynamics for a fixed number of agents in the population. If we continue the above analysis for ever larger population sizes, predictable patterns appear, such as (for *D* > 0):

- The vertices act as sinks, but the number of points attracted to them depends on the BCI.
- If the number of agents is even, the midpoint of the edges is accessible and acts as a source (for other points on the edge).
- If the number of agents is a multiple of three, the midpoint of each of the faces is accessible and acts as a source (for *D* = 1).
- If the number of agents is a multiple of four, the midpoint of the entire tetrahedron is accessible and acts as a source.

This suggests a different way of studying the opinion dynamics: instead of considering populations with a particular population size, one can consider populations in general and ask, for each possible opinion, which *fraction* of a population holds the opinion (this will be called the *density* the opinion has in the population). This in turn allows one to derive the above rules immediately, without the need for considering a large number of different population sizes. With the density-based information, one can still draw conclusions for particular population sizes. For instance, if 100% of the agents hold the same opinion, that represents a consensus (point at a vertex), which can occur in populations with any number of agents (*N* = 1, 2, 3, …). And if 50% of the agents hold one theory and 50% hold the other theory, there is a tie between two theories (midpoint of an edge); this can occur only in even-numbered populations (*N* = 2, 4, 6, …). In the next section, we consider such opinion densities. But first, we give a probabilistic interpretation concerning the results of the dynamics on the OPS.

### 4.2. Probabilistic interpretation of the OPS

We can give a probabilistic interpretation of the previous results. For instance, we may be interested in the probability that the agents in the population reach a consensus on a particular theory. If we assume that each initial opinion profile is equally likely (uniform prior probability), then the probability of reaching consensus on a particular theory is equal to the number of opinion profiles in the basin of this consensus position divided by the total number of points in the OPS. (For a non-uniform prior probability, we may compute a similar fraction based on weighted sums).

For *M* = 1 and *N* = 2, there are 10 points in the OPS and there is no dynamics, so the only way the population can end up in a consensus is by already starting out from that opinion profile. Hence, the probability of reaching consensus on a particular theory is 1/10. (The total probability of reaching a consensus is 4/10.) For *N* = 3, there are 20 points in the OPS. For *D* = 0, there is no dynamics, so the probability of reaching consensus on a particular theory is 1/20. (The total probability of reaching a consensus is 4/20, or 1/5.) For *D* = 1, two additional opinion profiles evolve toward each consensus position, so each basin consists of three points and the probability of reaching consensus on a particular theory is 3/20. (The total probability of reaching a consensus is 12/20, or 3/5.) For *D* = 2, each basin consists of four points and the probability of reaching consensus on a particular theory is 4/20, or 1/5. (The total probability of reaching a consensus is 16/20, or 4/5.) Given the nature of our update rule (EHK), it is not surprising that we find larger BCIs (larger values for *D*) to correspond with higher probabilities of reaching a consensus.

In our previous paper (Wenmackers et al., 2012), we only considered the probability that an agent, who starts from a consistent theory, updates to the inconsistent theory. For *M* = 1, this probability is zero. In general, there are $\frac{(N+t_{M}-2)!}{N!(t_{M}-2)!}$ opinion profiles in which no agent adheres to the inconsistent theory (i.e., the (hyper-)tetrahedral number of order *N* + 1 in *t*_*M*_ − 2 dimensions, or the multiset coefficient of choosing *N* times with repetition out of *t*_*M*_ − 1 options). For *M* = 1, these inconsistency-free opinion profiles are represented on a single face of the tetrahedral OPS—the face which has as its vertices each consensus on one of three consistent theories—and none of these evolve to consensus on the inconsistent theory. To investigate the phenomenon of consistent-to-inconsistent updating, we have to consider cases with larger values of *M*, as we did in our previous study (in which we assumed a uniform prior, not over all anonymous opinion profiles, but over the non-anonymous opinion profiles in which no agent adheres to the inconsistent theory).

### 4.3. Key points

An (anonymous) opinion profile specifies the number of agents that holds each of the theories. So, an opinion profile consists of 2^*t_M_*^ numbers that add up to *N*, the total number of agents in the population. We consider the space of all possible opinion profiles, the OPS. The dynamics on this space shows the group-level or aggregate effect of the individual updating by the rule introduced in the previous section. Some opinion profiles act as fixed points: once the population reaches such a state, there is no further dynamics. Consensus positions are stable fixed points, which “attract” nearby opinion profiles; equally balanced (or polarized) opinion profiles are unstable fixed points, which “push away” nearby opinion profiles. By counting states in the OPS and assigning priors probabilities to initial opinion profiles, we can give a probabilistic interpretation to the results. The analysis in terms of an OPS requires the choice of a particular population size, *N*; in the next section, we follow a slightly different approach that does not require this.

## 5. Opinion density space

To simplify the analysis, we leave the number *N* of agents open and represent all possible opinion profiles (for arbitrary *N*) simultaneously, using the opinion density space (ODS). For a given opinion profile $\vec{n}$, the corresponding opinion density $\vec{d}$ can be found via normalization, that is, division by the number *N* of agents: $\vec{d}$ = $\vec{n}$/*N*. Like $\vec{n}$, $\vec{d}$ is a vector with *t*_*M*_ components. We represented the components of a particular opinion profile $\vec{n}$ between angle brackets, 〈…〉; although confusion is unlikely, we will represent the components of an opinion density $\vec{d}$ between round brackets, (…). The opinion density coordinates can be viewed as barycentric coordinates, specifying which *fraction* of the agents adheres to each theory^6^.

Another way of looking at the transition from OPS to ODS is as follows: we can track the dynamics for a large set of different population sizes and represent the accumulated data in a single tetrahedral grid. In the limit where we combine the OPSs for all (infinitely many) finite population sizes, this accumulative OPS becomes continuous instead of a discrete grid. Hence, the ODS is a continuous space in *t*_*M*_ − 1 dimensions. (There are *t*_*M*_ components of the opinion density vector, which are fractions that sum to 1, so there remain *t*_*M*_ − 1 degrees of freedom).

To visualize the ODS, we have written an additional program in Object Pascal. Although the ODS represents a continuous space, numerical methods require it to be discretized, such that the program only encounters density vectors which have four rational indices. By multiplying the four rational indices of an opinion profile by their least common denominator, we compute an opinion profile that is representative of that density. The evolution of this profile is computed as before. The numerical result is indicated by means of colors (as explained below).

### 5.1. Results: dynamics on the opinion density space

We consider the ODS equipped with (EHK) as update rule (for particular values of *D*) as a continuous dynamical system.

As before, we focus on the case with *M* = 1. In this case, opinion densities have four digits, which are fractions that sum to 1, so there remain three degrees of freedom. Hence, these opinion densities can be represented using barycentric coordinates in a three-dimensional tetrahedron (inside the volume as well as on the surface). At the four vertices of the tetrahedral ODS for *M* = 1, we find the opinion profiles that have all their weight concentrated on a single theory, corresponding to populations in which all the agents agree on the same theory (consensus). On the edges of the tetrahedron, we find populations in which only two of the four theories are represented (the other two having density zero). On the faces of the tetrahedron, we find populations in which one of the four theories is not represented. Inside the volume of the tetrahedron, in each population there is at least one agent for each theory, so none of the density components is zero.

Also similar as before, we only represent a single face of the tetrahedral ODS: the triangle with vertices at (0, 0, 0, 1), (0, 0, 1, 0), and (1, 0, 0, 0), with the “double” edge at the bottom. Within this triangle, all opinion profiles have zero density at the second position: there are no agents that hold the theory 01.

**Figure 6:**
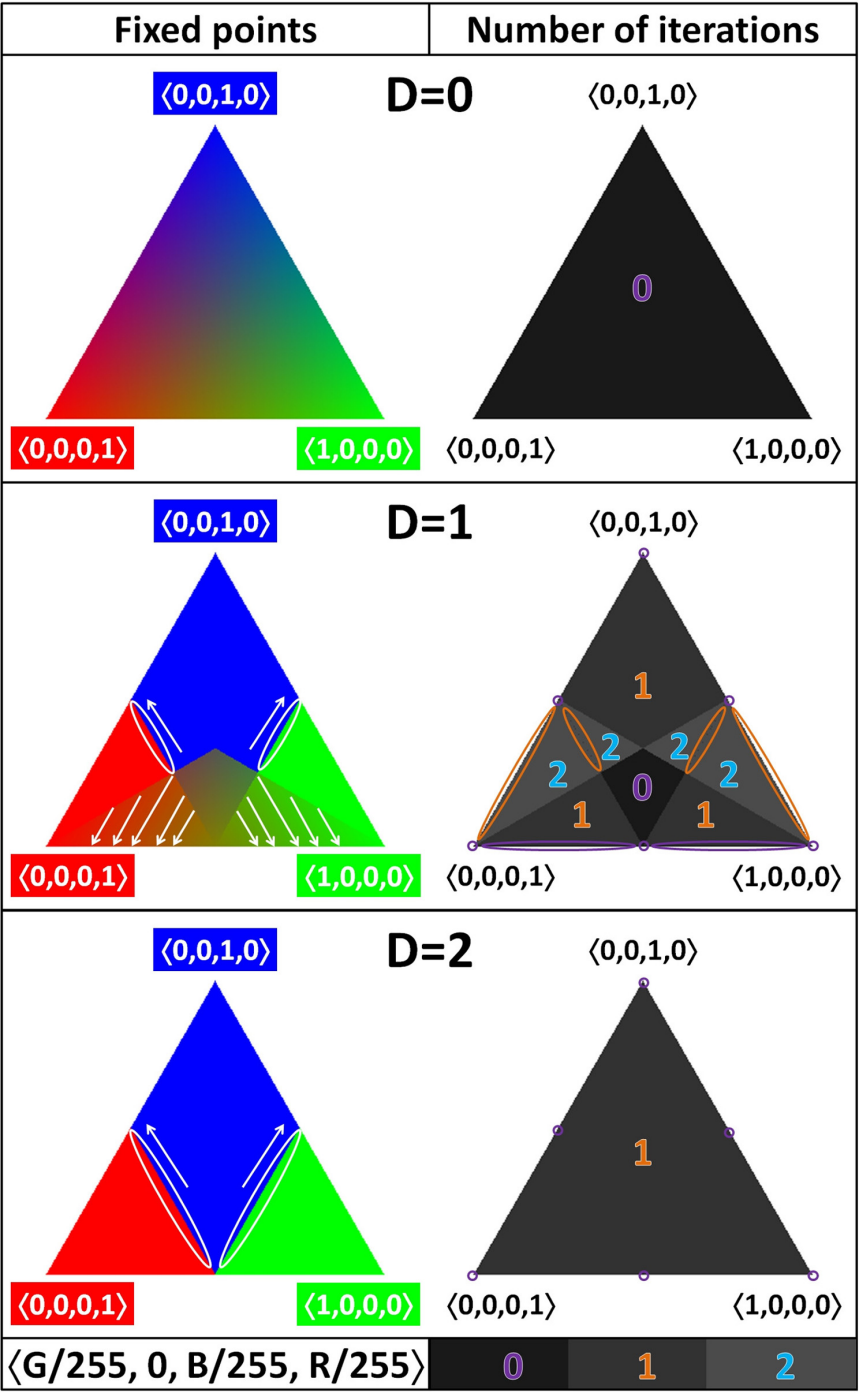
**Opinion dynamics for *M* = 1: one face of the opinion density space (ODS)**. On the left-hand side, each position in the ODS is colored depending on its final state (see main text for details); smaller features are indicated with white ellipses and arrows. On the right-hand side, each position in the ODS is given a gray-scale value depending on how many rounds of updating are required for it to reach its final state; smaller features are indicated with ellipses (purple for zero, orange for one, and blue for two). The value of the BCI threshold is varied: top row *D* = 0 (minimal), middle row *D* = 1 (intermediate), and bottom row *D* = 2 (maximal).

For each position in the chosen triangle, we compute the (normalized) opinion profile that it will ultimately evolve to. We represent this by a color. Specifically, the color (*R, G, B*) (with *R, G, B* ∈ {0, …, 255}) indicates that the opinion profile at that position will evolve to the opinion profile with barycentric coordinates equal to (*G*/255, 0, *B*/255, *R*/255). For instance, the redder a point, the larger the fraction of agents that will finally adhere to the inconsistent theory, 00. The results depend on the threshold value *D* and are presented at the left-hand side of Figure 6. For each point, we also indicate after how many steps the final state is reached. We represent this with a gray-scale on the right-hand side of Figure 6.

The results for *D* = 0 are trivial: the agents do not take the opinions of others into account, so there is no dynamics. On the right-hand side of Figure 6, we see that all the positions have the color corresponding to the initial opinion profile. At the left-hand side of Figure 6, we see that zero steps are required to reach the final state. Both observations confirm that all opinion profiles are fixed points. Because there is no dynamics, it is a situation of indifferent equilibrium. (This image is still helpful, because—due to the absence of dynamics—each point in it is colored based on its *own* coordinates, which can be used as a key to interpret the representation of the results with dynamics.)

The results for *D* = 2, the maximal threshold value in the case of *M* = 1, do show dynamics. In the colored image, we see clear evidence that a “double” edge of the tetrahedron was positioned at the bottom: it leads to a bilateral symmetry of the pattern. There are six fixed points. The three consensus positions at the vertices are fixed points, which act as sinks for large portions of the face. The three positions halfway along the edges are fixed points as well. Those on the “single” edges each attract opinion profiles from a line in the triangle; the fixed point on the “double” edge acts as a sink. The gray-scale image confirms these findings: the six fixed points do not require any iterations, whereas the others settle after just one update.

Intermediate values for *D* tend to lead to more complex and interesting behavior. This general trend holds up even for *M* = 1, although there is only one intermediate value: *D* = 1. The bilateral symmetry (and lack of additional symmetry), already observed for *D* = 2, is present here, too, but both the color and the gray-scale image show further features. There are fewer fixed points than for *D* = 0, but more than for *D* = 2: there is a kite-shaped region of fixed points (indifferent equilibrium), and the “double” edge consists of fixed points, all of which act as sinks for a line in the triangle. Moreover, this is the only case with *M* = 1 for which some initial opinion profiles require two rounds of updating to arrive at the final state.

Recall that for a fixed number of agents, not all points of the continuous ODS are accessible. Once you have computed the opinion dynamics for the ODS, you can use the results to construct the dynamics on an OPS for a fixed number of agents, *N*, by locating a density that is accessible for the *N* of interest and using the color of that point to determine to which opinion profile it will evolve. (In fact, the results on OPSs in the previous figures do already use the same color convention as that used for the ODS).

### 5.2. Probabilistic interpretation of the ODS

Similarly to the discussion of the OPS results, we also give a probabilistic interpretation of the results concerning the ODS. If we assume that each initial opinion profile is equally likely (uniform prior probability), then the probability of reaching consensus on a particular theory is equal to the volume of the basin associated with this consensus position divided by the total volume of the OPS. At least, this fraction expresses the limit probability associated with an infinite population size, in which the relative importance of special points (unstable equilibria) is vanishingly small.

**Figure 7:**
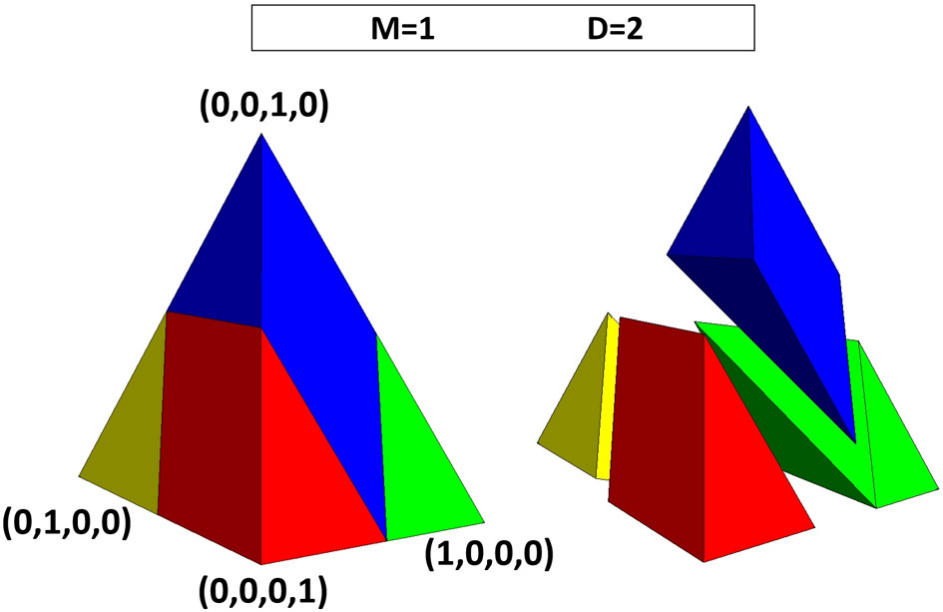
**Shape of the basins in the opinion density space (ODS) for *M* = 1 and *D* = 2**. Left-hand side: three-dimensional view of ODS with the face shown in previous figures turned toward the right. Right-hand side: exploded view of the same ODS, showing the four basins separately. Each basin has the same shape (with five faces: one rhombus, two equilateral triangles, and—facing the interior of the ODS—two isosceles right triangles) and a volume that occupies one quarter of the tetrahedral ODS.

In Figure 7, we illustrate the four basins associated with the four consensus positions in the ODS of *M* = 1 and *D* = 2. Each basin has the same shape with five faces: two equilateral triangles and one rhombus that face the exterior of the ODS and two isosceles right triangles that face the interior of the ODS (see also Supplementary Material). The four basins have one common edge (at the interior, where the isosceles right triangles meet) that connects the midpoints of the two “double edges” of the tetrahedral ODS.

Since the four basins have the same shape and size and together fill the entire volume of the ODS, they each correspond to a relative volume of 1/4. Under the assumption of a uniform prior, the limit of the probability of arriving at a particular consensus for exceedingly large populations is 1/4 (*M* = 1 and *D* = 2). For maximal *D*, the limit probability of arriving at some consensus is 1. Under these conditions, the unstable equilibria on the edges of the basins are isolated points, lines, or areas, which have zero volume and thus zero probability.

In particular, in the infinite population limit there is a probability of 1/4 of arriving at the inconsistent theory. However, if we only consider opinion densities where the inconsistent theory initially has zero density (which are all represented at the a single face of the ODS), the probability of evolving to an opinion profile with a non-zero density at the inconsistent theory (let alone unit density at this position) is zero (at least for *M* = 1).

### 5.3. Key points

Whereas the discrete OPS depends on a particular population size, *N*, the continuous ODS represents the density of theories in populations of arbitrary size. By considering volumes in the ODS and assigning a prior probability distribution to initial opinion profiles, we can give a probabilistic interpretation to the results, which serve as a good approximation for very large population sizes, but does not apply to small groups. We observe that even if special points (such as stable fixed points) make up a small portion of the ODS, these points tend to be represented in small populations (causing the dynamics to end after few rounds of updating).

## 6. General discussion

Due to social updating, an agent who starts out with a consistent theory about the world may arrive at the inconsistent theory. Even if maintaining consistency at all times is too demanding for non-ideal beings to qualify as a necessary condition for rationality (Cherniak, 1986), it is presumably something that rational beings should aim for. This may suggest that social updating is a vice, from the perspective of rationality. However, in our first study (Wenmackers et al., 2012) we computed the probability for an agent to update to the inconsistent theory and found it to be non-zero, but relatively small (lower than 2%); moreover, it can be made arbitrarily low by strategically varying the model parameters.

Our current study of the opinion dynamics on the belief space reveals another virtue of the social updating process: even if an agent starts out at the inconsistent theory, the agent's opinion may change—to one of the consistent theories—due to the social update rule. This could already be seen on the basis of Example 3.2, but the results depicted in Figure 6 give more systematic information in this respect: except for the rightmost edge and its two vertices, all the opinion profiles in the presented face of the tetrahedron contain at least one agent who starts out at the inconsistent theory. Nevertheless, when there is any dynamics at all, many of these opinion profiles evolve to different profiles, some of which have no agents at the inconsistent theory. This is true, in particular, for all the opinion profiles in the blue and green areas, which act as basins for consensus positions on consistent theories.

We have given a probabilistic interpretation of the results on the belief space (OPS and ODS). We have seen that in the limit for an infinite population size and for large BCIs (*D* = *w_M_*), the relative importance of unstable equilibria vanishes. For *M* = 1 and *D* = 2, the probability of arriving at a population-wide consensus on some theory is unity. In particular, the probability of arriving at a population-wide consensus on the inconsistent theory is 1/4. Once the agents reach consensus on the inconsistent theory, there will be no further dynamics, because all consensus positions are fixed points. Hence, this result may be regarded as a worst case. However, this case study is highly unrealistic for (at least) three reasons.

First, the assumption of a uniform prior on the opinion profiles does not apply to real cases. Observe that if the agents were to pick out their initial theory at random, the distribution of initial anonymous opinion profiles would be higher around the center of the belief space. (For larger populations, there are more combinations of individual theories that lead to an anonymous opinion profile, in which all theories are represented almost evenly.) More importantly, however, we do *not* expect the agents to adopt an initial theory at random but rather to possess some prior knowledge, such that the distribution of their initial theories is clustered around the true theory (which is necessarily a consistent one). Hence we also expect a preferential position of the opinion profiles in a region around consensus on the true theory. For this reason, investigation of a more complex model, based on a variant of our current update rule (EHK), but including evidence-gathering as well as social updating, is high on our to-do list.

Second, in many practical situations relevant population sizes tend to be small (just think of the last meeting you attended), such that the infinite population limit does not apply well to them. In smaller populations, the relative importance of unstable equilibria (which do not lead to consensus) is more pronounced.

Third, modeling belief states as theories of the world only has practical relevance when *M* > 1, for which the relative size of the basins associated with consensus positions decreases rapidly (as 1/*t_M_*).

For all these reasons, we estimate the probability of arriving at a consensus on the inconsistent theory to be very small in a realistic setting—in any case well below 1/4.

The mechanism for social updating may also be criticized in the following way. If agents' belief states are theories, their beliefs are closed under the consequence relation. So, illustrating with theories for the case of *M* = 1 (cf. Example 3.2), an agent whose belief state is characterized by the string 1100 is supposed to believe also the propositions coded as 1110, 1101, and 1111. This is not reflected in our current update rule (EHK) and suggests an asymmetric composition of the peer group: for *M* = 1 and *D* = 1, an agent *A* with theory 1111 and an agent *B* with theory 1100 are not each other's peers according to our current model. However, agent *B* also ought to believe *A*'s theory, but not vice versa. We may now suggest an alternative way of determining an agent's peer group: by taking into account also those agents that hold a theory which is within distance *D* of at least one of the consequences of the first agent's theory. Doing so would help to protect agents against updating to the inconsistent theory. However, it also introduces a preference for less informative theories, so it may hamper the agents' chances of finding the (strongest) true theory. Hence, this is a case where different epistemic goals (rationality versus finding the truth) are in direct conflict with each other and selecting the optimal normative model seems to require meta-norms of rationality.

In our previous work (Wenmackers et al., 2012), we have considered the probability of arriving at an opinion profile in which at least one agent adheres to the inconsistent theory, starting out from an opinion profile without any such agent (and assuming a uniform prior over these anonymous profiles). We found this probability to be zero for *M* = 1. This finding is confirmed in the current study. Nevertheless, by studying the dynamical space in general, we have observed certain trends that help to explain the previously obtained results for the probability of consistent-to-inconsistent updating.

For *M* = 2, the probability that an agent will arrive at the inconsistent theory, in a population where none have adopted this theory, is non-zero (provided that *D* > 0 and *N* > 2). In our previous work, we observed that this probability decreases when more independent issues are considered (that is, when *M* increases beyond 2). We are now in a better position to explain the—essentially combinatorial—mechanism behind this finding. Although we have not presented cross-sections for the higher-dimensional case, we can give a qualitative discussion of cases with *M* > 1. As *M* increases, the belief space becomes higher-dimensional (*t*_*M*_ − 1) and the basin that is attracted by the sink corresponding to consensus on the inconsistency becomes a smaller fraction of its total (hyper-)volume (equal to 1/*t_M_* for *D* = *w_M_*). This corresponds to the observation in our previous study that the probability of updating to the inconsistent theory is lowered by forming theories over more independent issues (higher *M*). For a larger number of agents (higher *N*), the dimensions of the belief space remain the same, but the opinion profile has access to more points of this space. As a result, the probability of consensus on the inconsistent theory is lower, too; this is in line with the earlier findings as well.

For belief spaces with a fixed number of agents (with *M* = 1 and *D* > 0), we observed that if the number of agents is even, the midpoint of the edges is accessible and acts as a source (in respect to other points on the edge). This is confirmed by our study of the ODS: the midpoint of an edge belongs to a line separating two or three basins. In the ODS, it also becomes clear that the midpoint on a “single” edge acts as a sink for points from the line between this midpoint and the midpoint of a “double” edge (half of the line for *D* = 1, all of it for *D* = 2). Moreover, if the number of agents is a multiple of four, the midpoint of the entire tetrahedron is accessible and acts as a source. In contrast, if the number of agents is a multiple of three, the midpoint of each of the faces is accessible and acts as a source (for *D* = 1). So, in the case of an even number of agents, there are more fixed points than in the case of an odd number of agents. Taken together, these effects explain the “even–odd wobble” in our previous study: the observation that agents have a lower probability of updating to the inconsistent theory in an even-numbered population than in an odd-numbered population of similar size.

Moreover, for fixed *M*, there is a limited number of these special points, whereas the total number of accessible points in the belief space rises fast when the number of agents, *N*, increases. Consequently, the number of these special points as compared to the total number of opinion profiles in the hyper-volume decreases when *N* increases, which explains the attenuation of the wobble for larger populations. If we consider (a face of) the ODS for *M* = 1 and *D* > 0 (cf. Figure 6), we see that the majority of opinion densities belong to some basin that is attracted to a sink. However, most of the points that are accessible in the OPS for a relatively small population size do not belong to these basins. Hence, small populations have a relatively high probability of producing delicately balanced opinion profiles, which tend to act as unstable equilibria (sources) and do not lead to full consensus.

Additionally, as the number *M* of propositions increases, the dimensionality of the belief space increases, as does the absolute number of these special points, but their number as compared to the possible points in the hyper-volume decreases. This explains the earlier observed decrease in the maximal probability of updating to the inconsistent theory as *M* increases.

While the model studied in this paper is idealized in several respects, it is not completely unrealistic. Even if real agents do not generally compromise with their peers exactly in the way our artificial agents do, real agents do tend to influence each other's belief states, whether consciously or not. Idealized models can give information about such processes, much in the way in which the Ideal Gas Law gives information about the behavior of real gases. Also, there are several ways to make the model more realistic, for instance, as indicated earlier, by providing the agents with direct evidence about the truth, which in our model could be added as a driving force, directed toward a particular theory, or—equivalently—as an external potential directed toward one of the vertices of the ODS, corresponding to consensus on a theory with exactly one non-zero bit.

But even in its present, idealized form, the model we have studied demonstrates that there may be issues of rationality specifically arising from the way or ways we interact epistemically with fellow inquirers. We will be content if this sways some traditional (“individualistic”) epistemologists as well as some psychologists to take the social level into consideration in their studies of rationality. For the latter group, we note that already the current model suggests a number of seemingly worthwhile empirical studies, focusing on how real people influence one another's belief states, on which factors determine whether people regard someone as their peer (in the technical sense used here), and on whether whatever epistemic interactions take place in reality tend to aid the achievement of people's epistemic goals.

## Funding

Sylvia Wenmackers's work was financially supported by a Veni-grant from the Netherlands Research Organization (NWO project 639.031.244 “Inexactness in the exact sciences”).

### Conflict of interest statement

The authors declare that the research was conducted in the absence of any commercial or financial relationships that could be construed as a potential conflict of interest.

## Footnotes

^1^Psychologists have recently begun to explore connections between rationality and argumentation, which can also be regarded as an appreciation of the fact that the social bears on questions of rationality. For a particularly noteworthy contribution in this vein, see Mercier and Sperber (2011), which argues that reasoning evolved primarily for argumentative purposes. See in this connection various of the contributions to the 2012 *Thinking & Reasoning* special issue on argumentation.

^2^See Andler (2012) for a critical discussion of the wisdom of the crowds idea.

^3^In our previous work, we concentrated on the possibility of ending up at the inconsistent theory, because believing a contradiction is generally considered as irrational. As a referee remarked, if the entire epistemic community ends up at the tautological theory, which represents a complete lack of knowledge about the world, this may also be considered as a vicious result—although not irrational *per se*. Since the current study does not focus on a particular result, but represents the dynamics in general, our results concerning the probabilities of ending up at the inconsistent theory are equally informative about the probabilities of ending up at the tautological theory. Specifically, for reasons of symmetry, the probabilities of arriving at a consensus on the tautological theory are identical to those for arriving at the inconsistent theory. In our previous work (Wenmackers et al., 2012), we only calculated the probability for one agent or the entire community to update to the inconsistent theory starting from a population in which no agent held this opinion, but we would have obtained the same probability values for one agent or all agents to update to the tautological theory starting from a population in which no agent held this opinion. Still, this inversion is less well motivated: starting out with the tautological theory is not necessarily a bad thing; some agents in the community may initially lack any evidence about the world and try to arrive at a more informative theory through social updating.

^4^To forestall misunderstanding, it is worth mentioning that the word “Bounded” in “Bounded Confidence Interval” refers to the fact that the confidence intervals in the HK model have a lower and upper bound; in particular, the word is not meant to suggest any connection with Simon's notion of bounded rationality (see, e.g., Simon, 1955). The closest connection in the psychological literature is with the notion of confirmation bias, inasmuch as the BCI encompasses those agents whose opinions could be said to confirm to some extent one's own opinion.

^5^To avoid later disappointment, we note already at this juncture that, while we are introducing a general framework for representing theories, our own later investigations of this framework will focus on the *M* = 1 case.

^6^The notion of barycentric coordinates, which comes from geometry, may need some introduction. We first introduce the concept of a simplex. A two-dimensional simplex is a triangle: a figure with three vertices. In three dimensions, a simplex is a tetrahedron: a figure with four vertices. In general, in *k* dimensions, a simplex is a figure with *k* + 1 vertices. The ODS is a simplex with 2^*t_M_*^ = 4 vertices. Hence, for the simplest case with *M* = 1, we are dealing with a tetrahedral ODS in *k* = 2^*t_M_*^ − 1 = 3 dimensions. To indicate a particular point inside a *k*-dimensional simplex, one can use *k* Euclidean coordinates (belonging to *k* orthogonal axes), but for many applications it is more natural to use barycentric coordinates. The word “barycenter” refers to the center of mass, and barycentric coordinates indicate how much a point “gravitates” toward each of the vertices of the simplex. Since a *k* dimensional simplex has *k* + 1 vertices, it also has *k* + 1 barycentric coordinates, but since those coordinates are fractions that sum to unity, there are only *k* degrees of freedom. In three dimensions, a barycentric plot indicates the ratios of four quantities. The geometric center of a *k*-dimensional simplex is characterized by *k* + 1 barycentric coordinates that are all equal to 1/(*k* + 1). In general, points inside the (hyper-)volume of the simplex have barycentric coordinates that are all strictly positive. Points with one barycentric coordinate equal to unity and all the others equal to zero indicate a vertex position.
