# Supplementary material for: Rationality: a social-epistemology perspective
Source: Front Psychol. 2014 Jun 18;5:581. doi: 10.3389/fpsyg.2014.00581 (PMC4061898; doi:10.3389/fpsyg.2014.00581)
Supplement: Supplementary file 1 [file Presentation1.PPT]

## Slide 1
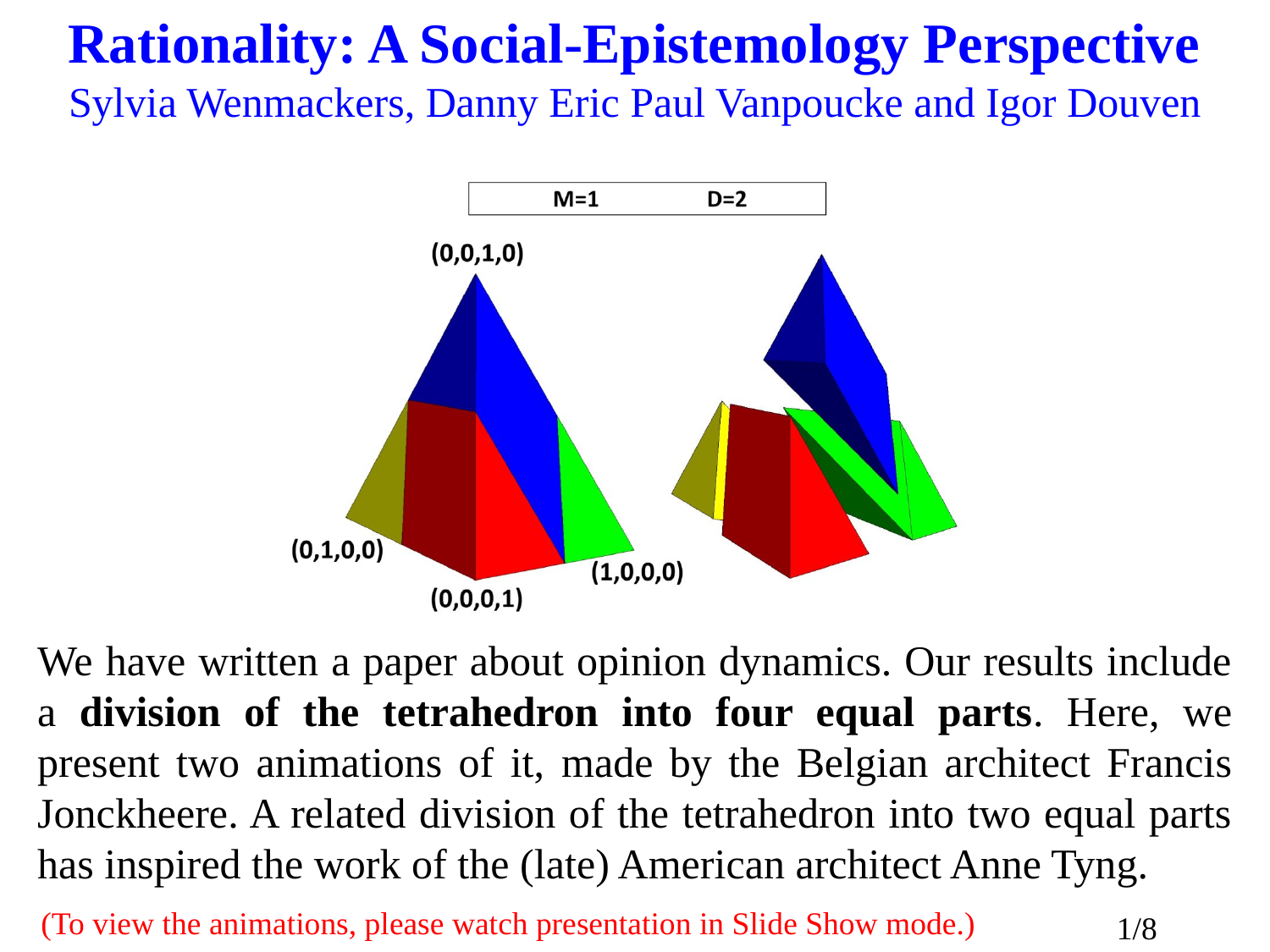

Rationality: A Social-Epistemology Perspective
Sylvia Wenmackers, Danny Eric Paul Vanpoucke and Igor Douven
We have written a paper about opinion dynamics. Our results include a division of the tetrahedron into four equal parts. Here, we present two animations of it, made by the Belgian architect Francis Jonckheere. A related division of the tetrahedron into two equal parts has inspired the work of the (late) American architect Anne Tyng.
(To view the animations, please watch presentation in Slide Show mode.)
<number>/8

## Slide 2
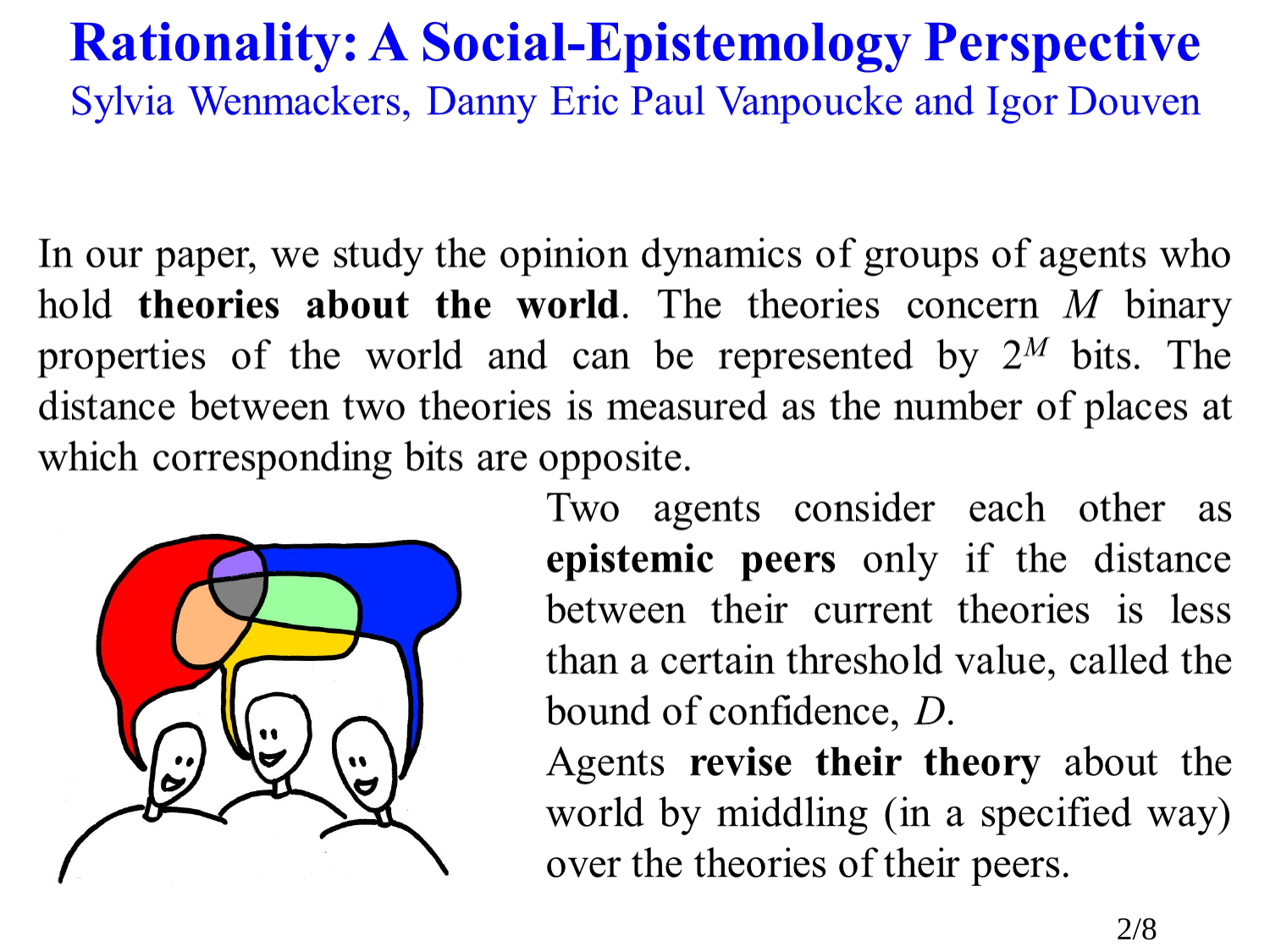

<number>/8

## Slide 3
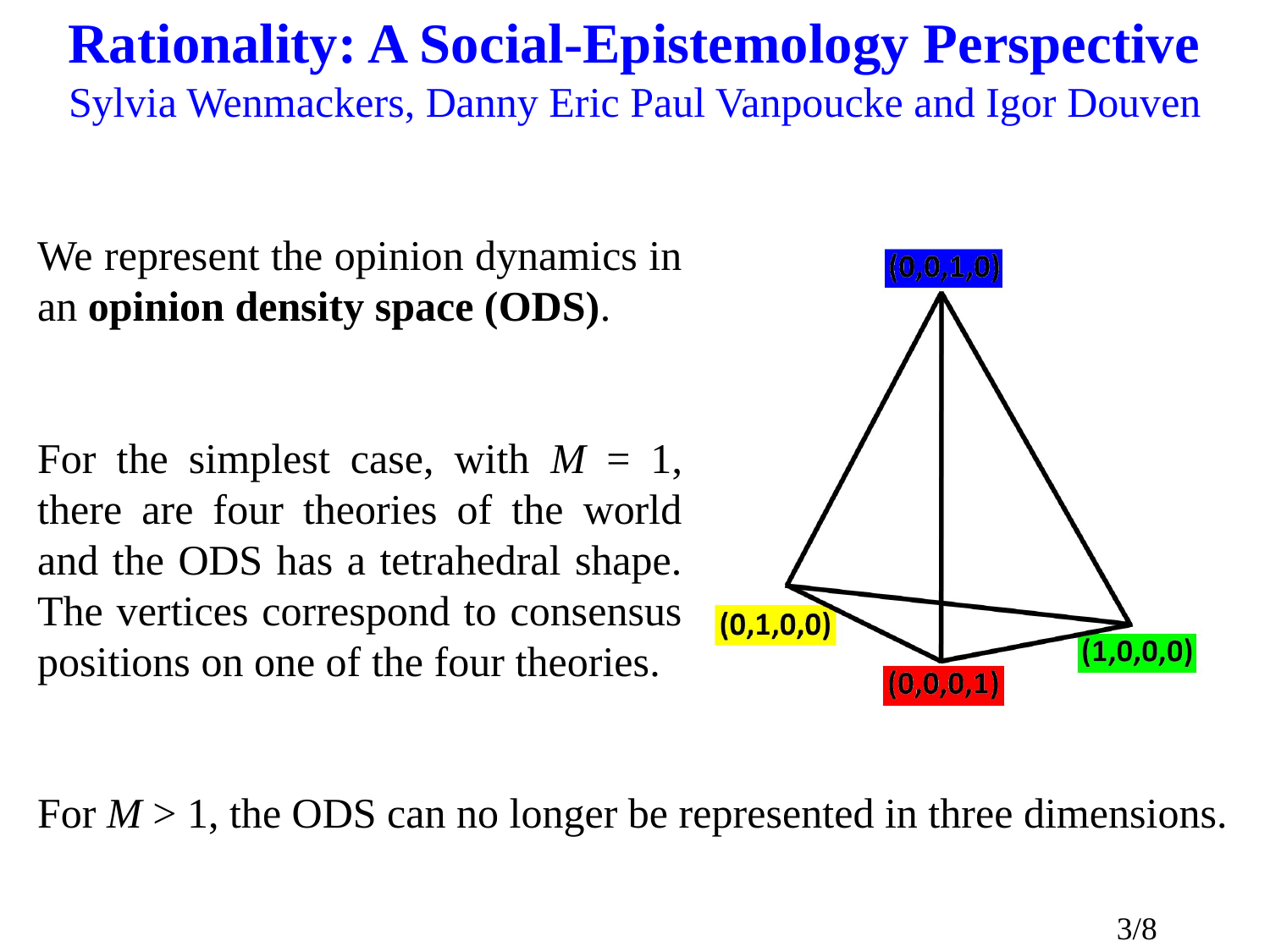

Rationality: A Social-Epistemology Perspective
Sylvia Wenmackers, Danny Eric Paul Vanpoucke and Igor Douven
For M > 1, the ODS can no longer be represented in three dimensions.
We represent the opinion dynamics in an opinion density space (ODS).
For the simplest case, with M = 1, there are four theories of the world and the ODS has a tetrahedral shape. The vertices correspond to consensus positions on one of the four theories.
<number>/8

## Slide 4
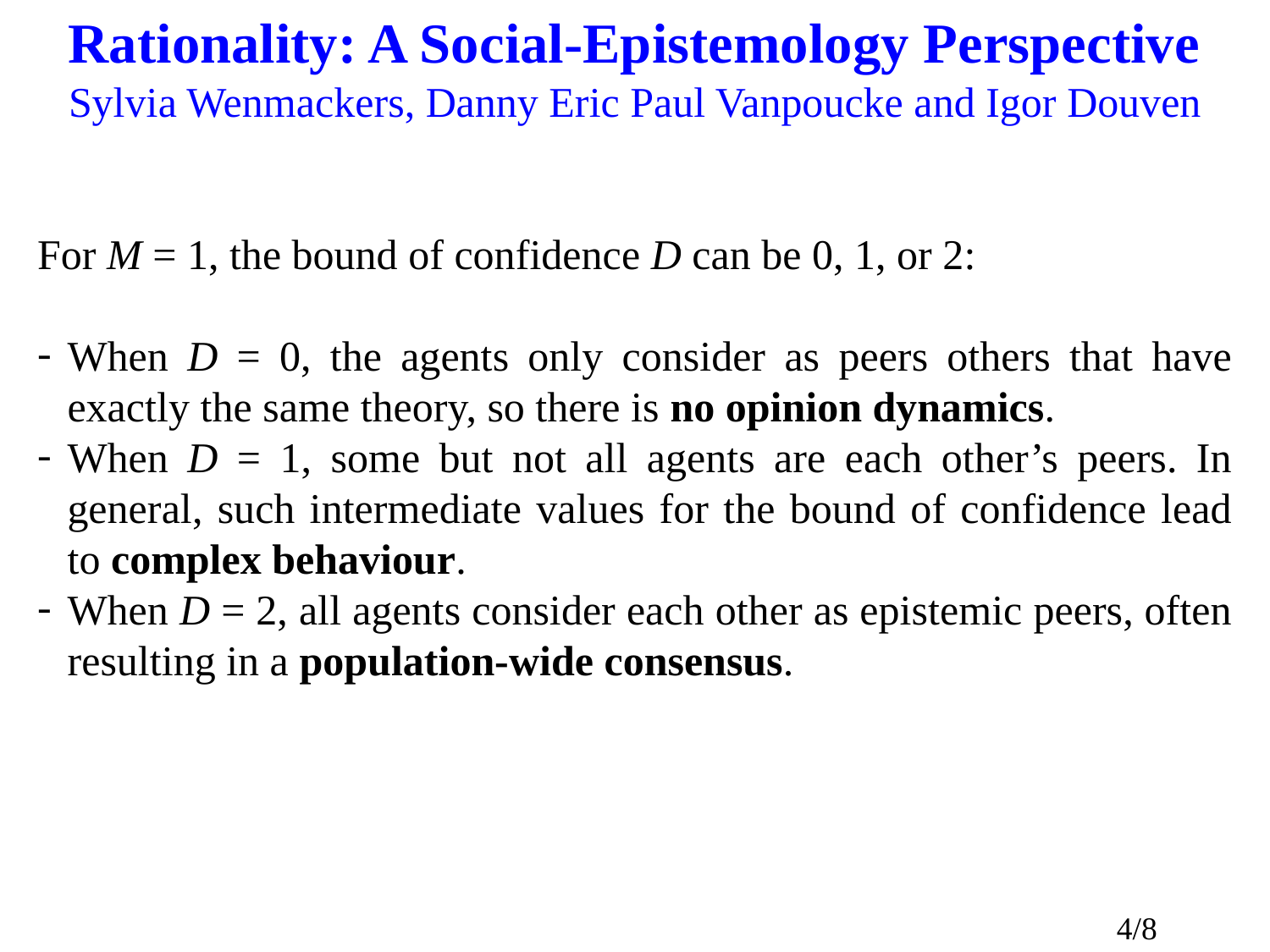

Rationality: A Social-Epistemology Perspective
Sylvia Wenmackers, Danny Eric Paul Vanpoucke and Igor Douven
For M = 1, the bound of confidence D can be 0, 1, or 2:
When D = 0, the agents only consider as peers others that have exactly the same theory, so there is no opinion dynamics.
When D = 1, some but not all agents are each other’s peers. In general, such intermediate values for the bound of confidence lead to complex behaviour.
When D = 2, all agents consider each other as epistemic peers, often resulting in a population-wide consensus.
<number>/8

## Slide 5
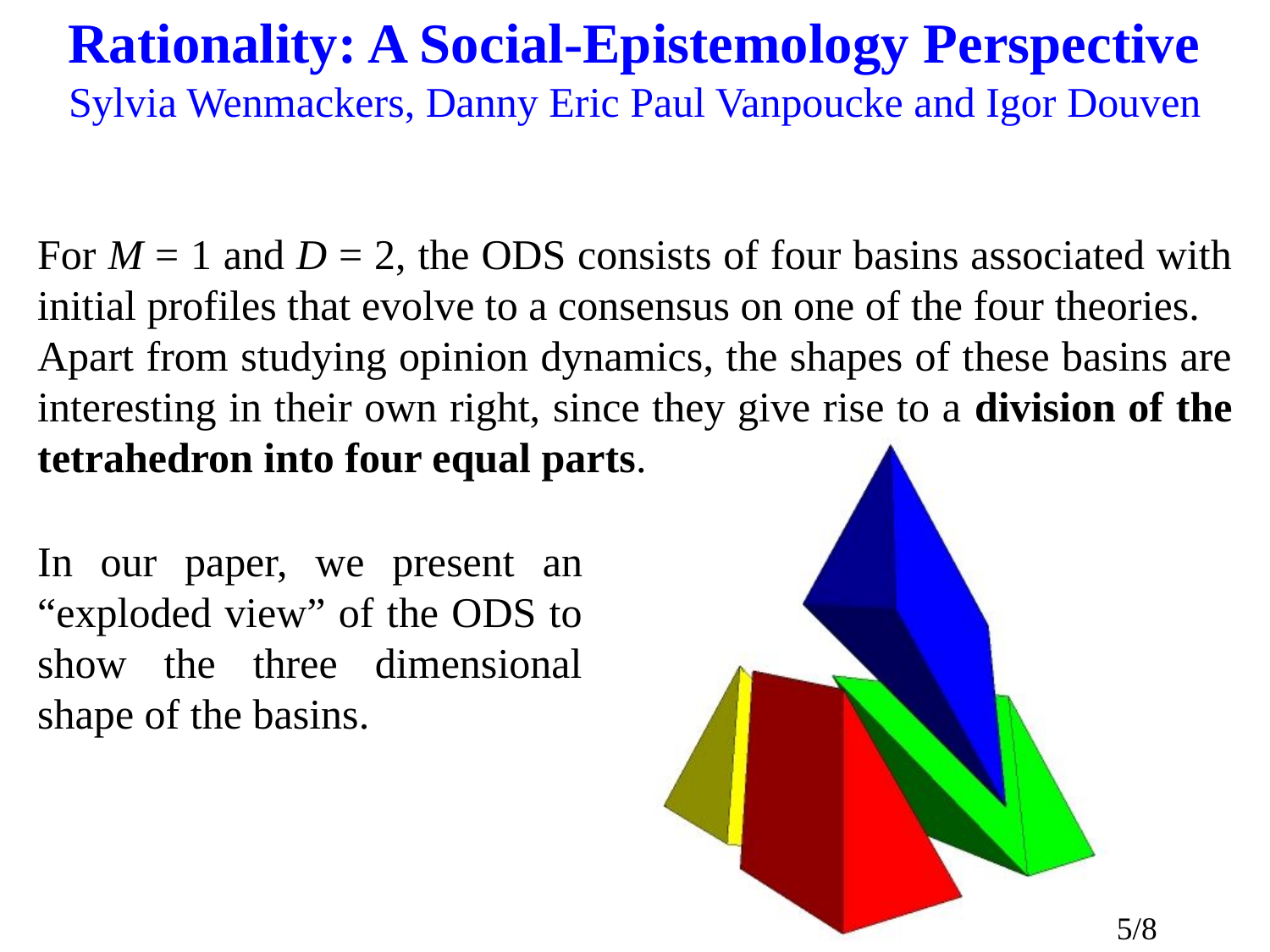

Rationality: A Social-Epistemology Perspective
Sylvia Wenmackers, Danny Eric Paul Vanpoucke and Igor Douven
For M = 1 and D = 2, the ODS consists of four basins associated with initial profiles that evolve to a consensus on one of the four theories.
Apart from studying opinion dynamics, the shapes of these basins are interesting in their own right, since they give rise to a division of the tetrahedron into four equal parts.
In our paper, we present an “exploded view” of the ODS to show the three dimensional shape of the basins.
<number>/8

## Slide 6
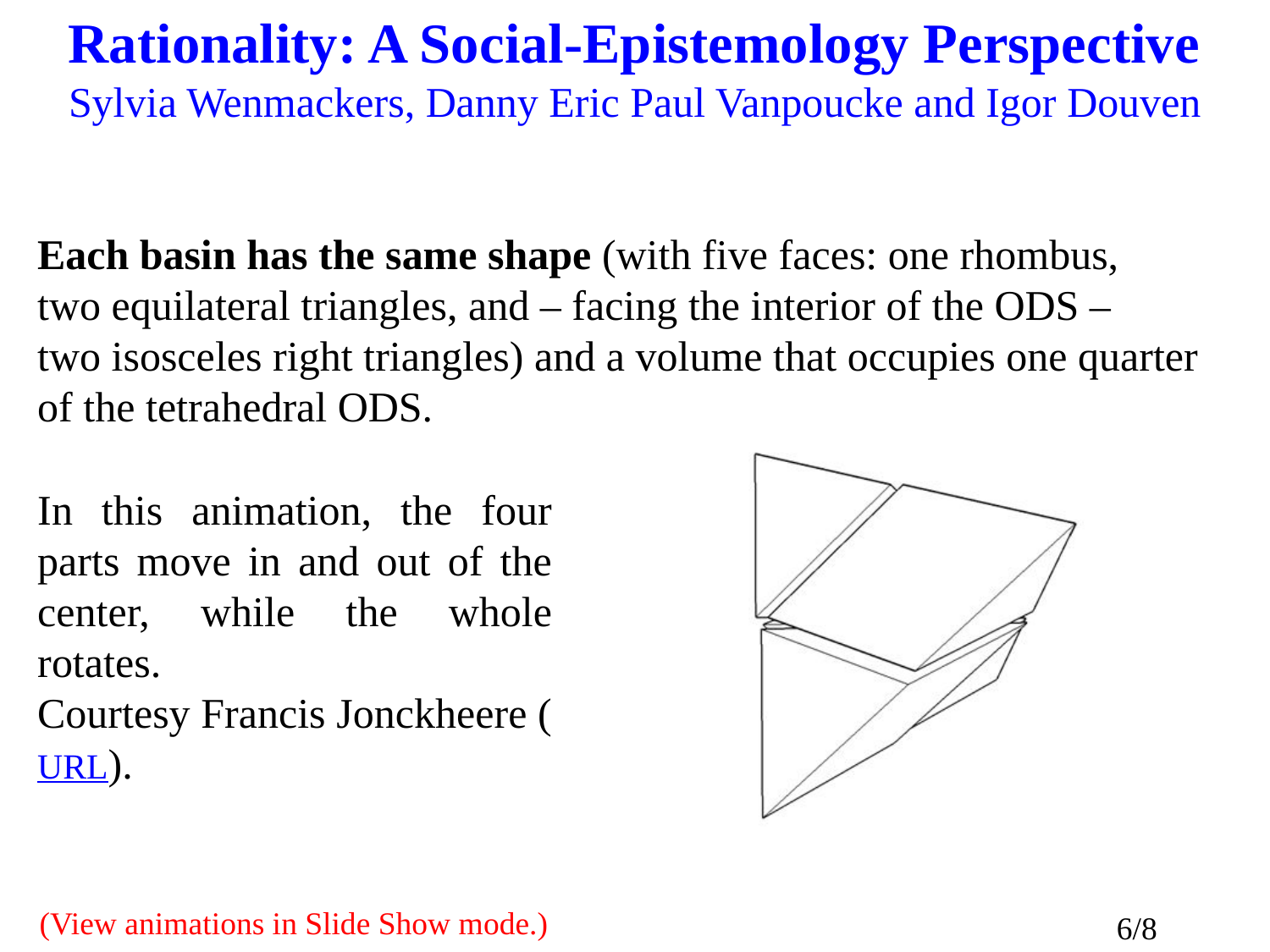

Rationality: A Social-Epistemology Perspective
Sylvia Wenmackers, Danny Eric Paul Vanpoucke and Igor Douven
Each basin has the same shape (with five faces: one rhombus,
two equilateral triangles, and – facing the interior of the ODS –
two isosceles right triangles) and a volume that occupies one quarter of the tetrahedral ODS.
In this animation, the four parts move in and out of the center, while the whole rotates.
Courtesy Francis Jonckheere (URL).
(View animations in Slide Show mode.)
<number>/8

## Slide 7
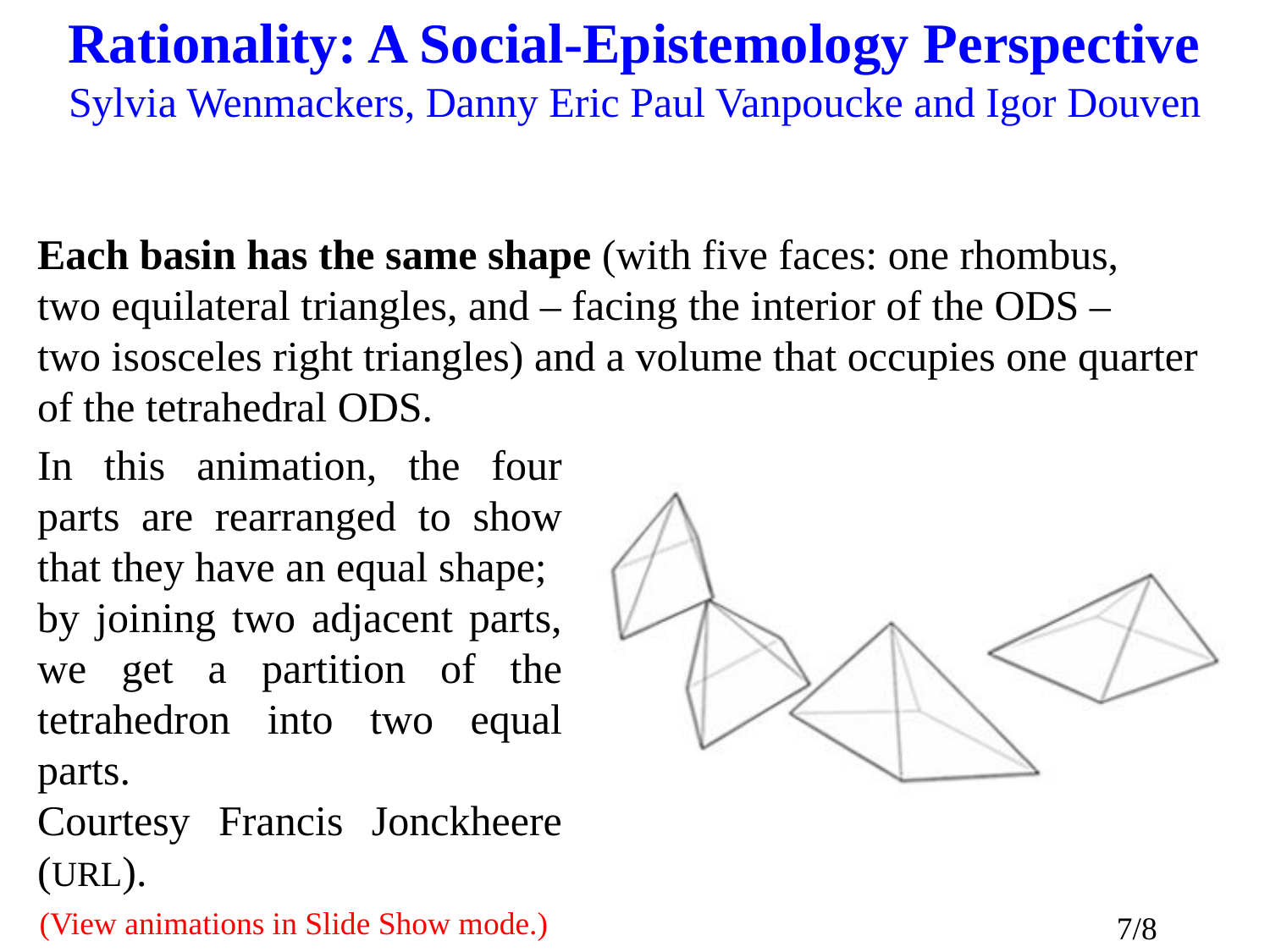

Rationality: A Social-Epistemology Perspective
Sylvia Wenmackers, Danny Eric Paul Vanpoucke and Igor Douven
Each basin has the same shape (with five faces: one rhombus,
two equilateral triangles, and – facing the interior of the ODS –
two isosceles right triangles) and a volume that occupies one quarter of the tetrahedral ODS.
In this animation, the four
parts are rearranged to show
that they have an equal shape;
by joining two adjacent parts,
we get a partition of the
tetrahedron into two equal
parts.
Courtesy Francis Jonckheere (URL).
(View animations in Slide Show mode.)
<number>/8

## Slide 8
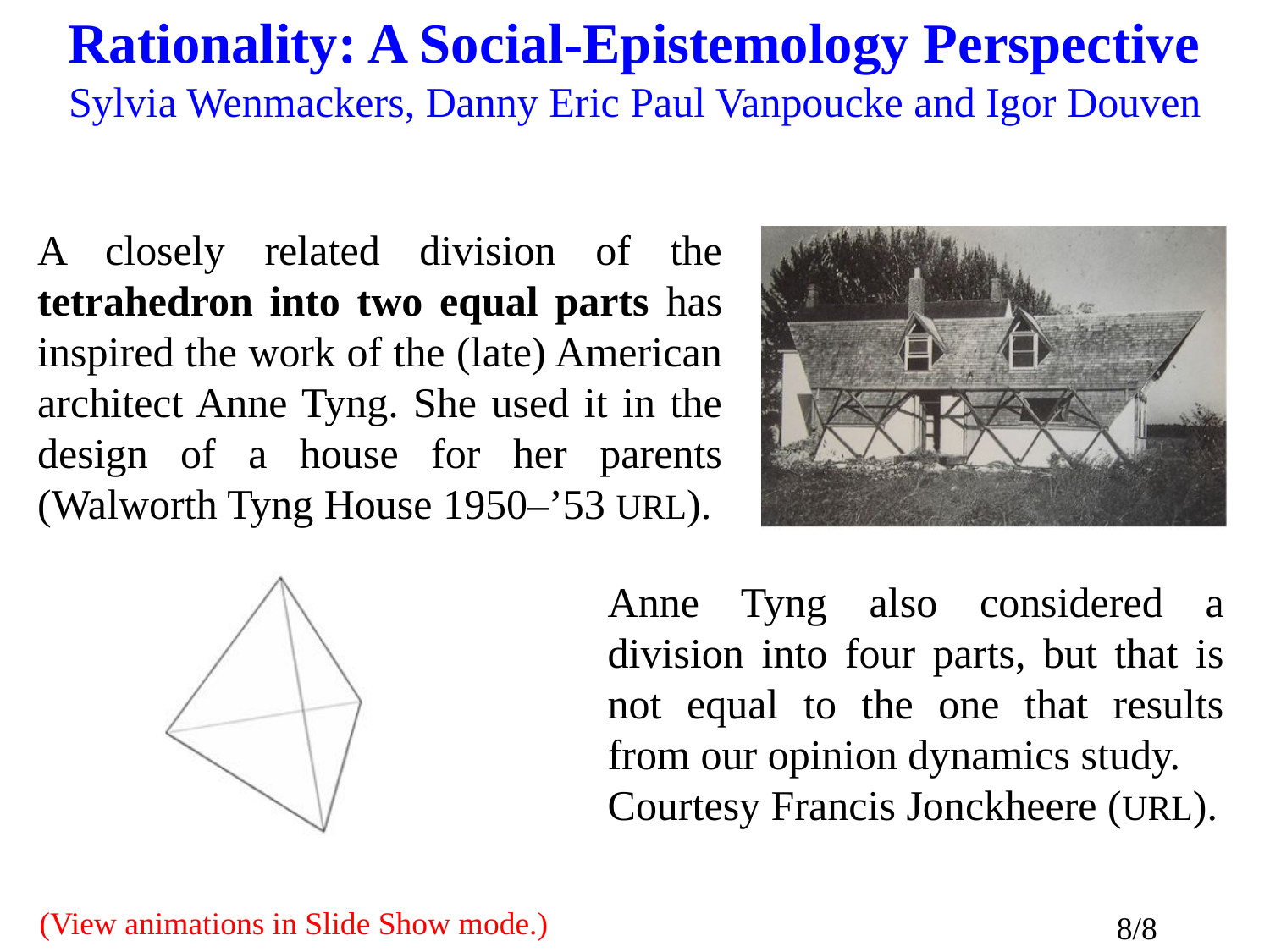

Rationality: A Social-Epistemology Perspective
Sylvia Wenmackers, Danny Eric Paul Vanpoucke and Igor Douven
A closely related division of the tetrahedron into two equal parts has inspired the work of the (late) American architect Anne Tyng. She used it in the design of a house for her parents (Walworth Tyng House 1950–’53 URL).
Anne Tyng also considered a division into four parts, but that is not equal to the one that results from our opinion dynamics study.
Courtesy Francis Jonckheere (URL).
(View animations in Slide Show mode.)
<number>/8
